# Shooting Mechanisms in Nature: A Systematic Review

**DOI:** 10.1371/journal.pone.0158277

**Published:** 2016-07-25

**Authors:** Aimée Sakes, Marleen van der Wiel, Paul W. J. Henselmans, Johan L. van Leeuwen, Dimitra Dodou, Paul Breedveld

**Affiliations:** 1 Department of Biomechanical Engineering, Delft University of Technology, Delft, the Netherlands; 2 Experimental Zoology Group, Wageningen Institute of Animal Sciences, Wageningen University, Wageningen, the Netherlands; The University of Wisconsin - Madison, UNITED STATES

## Abstract

**Background:**

In nature, shooting mechanisms are used for a variety of purposes, including prey capture, defense, and reproduction. This review offers insight into the working principles of shooting mechanisms in fungi, plants, and animals in the light of the specific functional demands that these mechanisms fulfill.

**Methods:**

We systematically searched the literature using Scopus and Web of Knowledge to retrieve articles about solid projectiles that either are produced in the body of the organism or belong to the body and undergo a ballistic phase. The shooting mechanisms were categorized based on the energy management prior to and during shooting.

**Results:**

Shooting mechanisms were identified with projectile masses ranging from 1·10^−9^ mg in spores of the fungal phyla Ascomycota and Zygomycota to approximately 10,300 mg for the ballistic tongue of the toad *Bufo alvarius*. The energy for shooting is generated through osmosis in fungi, plants, and animals or muscle contraction in animals. Osmosis can be induced by water condensation on the system (in fungi), or water absorption in the system (reaching critical pressures up to 15.4 atmospheres; observed in fungi, plants, and animals), or water evaporation from the system (reaching up to −197 atmospheres; observed in plants and fungi). The generated energy is stored as elastic (potential) energy in cell walls in fungi and plants and in elastic structures in animals, with two exceptions: (1) in the momentum catapult of Basidiomycota the energy is stored in a stalk (hilum) by compression of the spore and droplets and (2) in Sphagnum energy is mainly stored in compressed air. Finally, the stored energy is transformed into kinetic energy of the projectile using a catapult mechanism delivering up to 4,137 J/kg in the osmotic shooting mechanism in cnidarians and 1,269 J/kg in the muscle-powered appendage strike of the mantis shrimp *Odontodactylus scyllarus*. The launch accelerations range from 6.6*g* in the frog *Rana pipiens* to 5,413,000*g* in cnidarians, the launch velocities from 0.1 m/s in the fungal phylum Basidiomycota to 237 m/s in the mulberry *Morus alba*, and the launch distances from a few thousands of a millimeter in Basidiomycota to 60 m in the rainforest tree *Tetraberlinia moreliana*. The mass-specific power outputs range from 0.28 W/kg in the water evaporation mechanism in Basidiomycota to 1.97·10^9^ W/kg in cnidarians using water absorption as energy source.

**Discussion and conclusions:**

The magnitude of accelerations involved in shooting is generally scale-dependent with the smaller the systems, discharging the microscale projectiles, generating the highest accelerations. The mass-specific power output is also scale dependent, with smaller mechanisms being able to release the energy for shooting faster than larger mechanisms, whereas the mass-specific work delivered by the shooting mechanism is mostly independent of the scale of the shooting mechanism. Higher mass-specific work-values are observed in osmosis-powered shooting mechanisms (≤ 4,137 J/kg) when compared to muscle-powered mechanisms (≤ 1,269 J/kg). The achieved launch parameters acceleration, velocity, and distance, as well as the associated delivered power output and work, thus depend on the working principle and scale of the shooting mechanism.

## Introduction

In nature, shooting mechanisms are used for a variety of purposes including reproduction, prey capture, and defense. Shooting mechanisms evolved multiple times in a diversity of plant and fungal taxa, from the catapulting mechanisms in ferns, the water jet mechanisms in ascomycetes, and the air pressure gun of peat mosses (launching spores with accelerations up to 36,000*g* [1]) (*g* = magnitude of the gravitational acceleration [9.81 m/s2]), to the exploding seeds, fruits, and flowers of angiosperms (i.e. flowering plants). Similarly, fast movements occur in disparate animal groups, including stomatopods (marine crustaceans) that use a fast appendage strike to ambush prey, cnidarians that shoot stinging organelles at prey or foe with accelerations reaching 5,413,000*g* [2], and small chameleons that shoot their tongues with accelerations up to 264*g* (in the smallest specimens) to capture elusive prey [3]. Each of these highly effective shooting mechanisms fulfills specific functional demands and has evolved under the influence of natural selection [4]. This has resulted in several unique adaptations that can be linked to a range of successful adaptive radiations (see [5,6] for an example of adaptive radiation in lungless salamanders, family Plethodontidae).

Insight into the working principles of biological shooting mechanisms can provide important clues about how to design dedicated artificial shooting mechanisms, that could be used, for example, for puncturing biological tissues with high accuracy (needed in biopsies) and high-speed pick-and-place applications. Over the past few decades, the morphology and working principles of shooting mechanisms found in plants [7,8] and fungi [9,10] have been reviewed, but a comprehensive review on shooting mechanisms in animals and a comparative analysis of shooting mechanisms across kingdoms are still missing. Here we intend to fill this gap by providing a comparative overview of shooting mechanisms found in these taxa. We focus on the energy management prior to and during shooting, as a key element that enables the extreme performance of biological shooting mechanisms. Specifically, the identified shooting mechanisms are classified depending on how the energy is generated, stored, and transformed into kinetic energy of the projectile. As shooting mechanisms are found from micro- to macroscale, scaling effects will also be addressed. For this purpose, we will discuss the launch acceleration, velocity, distance, and direction, as well as the power and work delivered by the shooting mechanism per unit mass.

## Literature Search Method

### Search Strategy and Eligibility Criteria

We conducted two separate search queries: one for shooting mechanisms in plants and fungi, and one for animals, since the terminology used for plants and fungi differs from that in animal studies. The literature searches were performed in Scopus and the Web of Science Core Collection (last update: 05 May 2016). The full search queries and search strategies are provided in S1 Appendix. The PRISMA checklist is provided in S1 Checklist.

We only considered articles in the English language and focused the literature search on solid projectiles. Only shooting mechanisms in which the projectile is produced in the body (e.g. a spore or seed) or is part of the body (e.g. chameleon tongue) and undergoes a ballistic trajectory were included. We excluded the following systems: (1) Shooting mechanisms in which foreign objects are used as projectiles (occurring often in throwing actions). (2) Liquid and gas projectiles, such as the Archer fish that shoots down prey from overhanging foliage with a fast, forceful water shot [12] and the Bombardier beetle that uses a liquid venom for defense [13]. (3) Jumping and throwing actions, sometimes referred to as shooting (e.g. the catapult mechanisms used by froghopper to jump with accelerations of up to 408*g* [14]). (4) Mechanisms in which shooting is directly triggered by the environment, such as spore and seed launch by means of raindrop impact [15] and buzz-pollination in flowers, in which pollen are ejected by means of bumble bee vibrations [16,17]. (5) Single-cellular or subcellular shooting mechanisms. (6) Relatively slow (tongues in some frogs [18]) and fast (tentacle strike in squid [19]) extensions by muscular hydrostats that are not truly ballistic.

### Study Selection

The plants and fungi search yielded 290 and 233 articles in the Scopus and Web of Science databases, respectively, with 172 duplicates between the databases, resulting in 351 unique articles. The titles and abstracts of these articles were screened for relevance by two of the authors. The full text of a paper was assessed if both authors did not reject a title or abstract based on the eligibility criteria. Disagreements were resolved by discussion and consensus, which led to a selection of 48 articles. In addition, 20 articles were retrieved from the reference lists of these papers, resulting in 68 articles included in the review. For the full search strategy and study selection in plants and fungi see S1 Appendix and S1 Fig.

The animal search queries yielded 408 and 186 articles in the Scopus and Web of Science databases, respectively, with 143 duplicates between the two databases, resulting in 451 unique articles. Following the same protocol as described for the plants and fungi, a total of 51 articles were selected. Fourteen additional articles were retrieved from the reference lists, resulting in 65 articles included in this review. The full search strategy and study selection in animals is given in S1 Appendix and S2 Fig.

## Categorization

We based our categorization of the examined biological shooting mechanisms on the employed energy management, which typically involves (1) energy generation, (2) energy storage, and (3) energy transformation.

### Energy Generation

In all the identified shooting mechanisms in plants and fungi, energy for shooting is generated through osmosis (i.e. the diffusion of water through a semi-permeable membrane triggered by a change in concentration of osmolytes, that is, ions, sugars, and alcohols, in two neighboring solutions). To achieve osmosis, the osmolytes are actively transported through the cell membrane against a concentration gradient. This is accomplished by either transmembrane protein pumps (ATPases), which power the active transport by splitting adenosine triphosphate (ATP), or coupled transport pumps that use potential energy by exploitation of an electrochemical gradient. Depending on the direction of the water exchange, three osmosis-controlled mechanisms of energy generation can be distinguished: (1) water condensation on the outer surface of the shooting mechanism, (2) water absorption into the cells of the shooting mechanism, and (3) water evaporation from the cells of the shooting mechanism. Plants use only water absorption and water evaporation, whereas in fungi all three osmosis-controlled mechanisms occur. Some animals also use an osmosis-controlled water absorption mechanism, but the energy for shooting is most often generated by the contraction of muscle fibers located in the proximity to or even in the projectile. To achieve contraction, muscle fibers contain myofibrils, which have serially arranged contractile units, called sarcomeres (see [18] for the sliding-filament theory). The sarcomeres contain a lattice of actin and myosin filaments, which are able to slide along each other. When an impulse arrives at the neuromuscular junction, neurotransmitters (such as acetylcholine) are released, which in turn causes an action potential of the sarcolemma and ultimately the release of calcium ions (Ca^2+^) from the sarcoplasmic reticulum. The Ca^2+^ binds to Troponin C (a regulatory protein) on the actin filament, which then exposes the binding location for the myosin heads of the myosin filaments (with adenosine diphosphate (ADP) and inorganic phosphate bound to its nucleotide binding pocket). Hence, cross-bridges can be formed between the actin and myosin filament. Cross-bridges can make a mechanical power stroke at the expense of one ATP-bond per cycle. The release of ADP and inorganic phosphates from the myosin enables the myosin filaments to pull the actin filaments inwards, shortening the muscles. Finally, the binding of ATP to the myosin head allows it to break the cross-bridges with the actin filament.

### Energy Storage

The generated energy is stored in a medium as elastic energy (a form of potential energy) until it reaches a critical level or is released by a triggering mechanism. In plants and fungi, the energy for shooting is generally stored as elastic energy using pressure changes inside the cell that deform the cell wall. The absorption of water into the cell pushes the expandable plasma membrane against the rigid cell wall (also known as turgor pressure), resulting in a turgid cell, whereas the evaporation of water causes a negative pressure (with respect to ambient) inside the cell that pulls the plasma membrane from the cell wall, resulting in a plasmolyzed cell [8]. In two of the identified mechanisms, energy is not primarily stored in the cell wall: (1) in the momentum catapult of Basidiomycota, energy is stored in a stalk by an increase and shift in the center of mass of the spore [19] and (2) in *Sphagnum*, energy is mainly stored in pressurized air contained within a spherical capsule [1]. In animals, the energy for shooting is stored as elastic energy in collagen or other fibrous structures such as resilin. Storing energy in elastic structures can be advantageous over direct use of energy for muscle contraction, because elastic tissue structures can recoil much faster than muscles can shorten [20,21]. In other words, while contracting muscle tissues have a peak mass-specific power output of 373 W/kg in amphibians at 25°C [22] and 1,121 W/kg in quail flight muscle [23,24], elastic tissues can reach power outputs of, for example, 470,000 W/kg in mantis shrimps [25]. Furthermore, the mechanical properties of elastic tissues are less temperature-sensitive than muscle contraction, allowing the animals to use their launch mechanisms over a wide temperature range [26–28].

### Energy Release & Transformation

The stored energy is released and transformed to kinetic energy of the projectile. In plants, the elastic energy is released by the fracture of molecular bonds or cavitation inside the cytoplasm of the cells. Similar release mechanisms occur in fungi. An exception is found in the fungi genus *Sphaerobolus*, where the stored elastic energy is released by the eversion of a membrane [10]. In animals, the stored elastic energy for shooting is either released by eversion of the projectile itself in cnidarians [29], relaxation of collagen fibers in the ballistic tongues in chameleons [30] and lungless salamanders [31], or release of a latch in stomatopods [25]. Unfortunately, not all release mechanisms in animals are known or sufficiently understood, such as those of frogs that use a rapid jaw movement to project their tongue [16]. Finally, the stored energy is transferred to the projectile, which gains kinetic energy, using a specific catapult mechanism.

## Fungi

**Fig 1:**
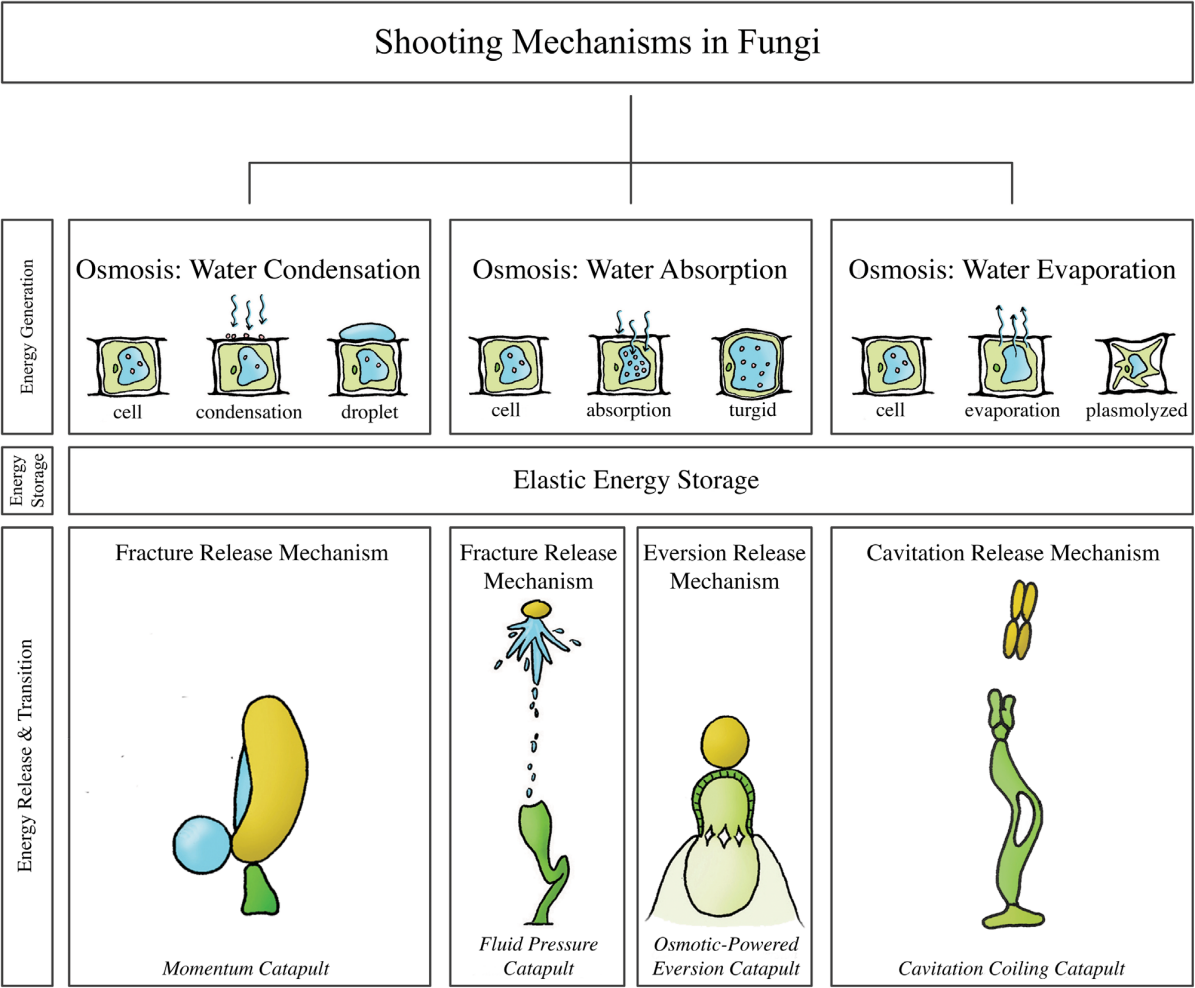
The structural categorization of the identified shooting mechanisms in fungi, allocated based on the energy management criteria discussed above. **Momentum catapult**: observed in the phylum Basidiomycota, schematic illustration of *Auricularia auricular*. **Fluid pressure catapult**: observed in the phylum Ascomycota and the genera *Pilobolus* and *Basidiobolus* of the phylum Zygomycota, schematic illustration of *Pilobolus kleinii*. **Osmotic-powered eversion catapult**: observed in the genus *Sphaerobolus*. **Cavitation coiling catapult**: observed in genera and species of the phylum Ascomycota and Basidiomycota, schematic illustration of *Zygophalia jamaicensis*.

Fig 1 illustrates the shooting mechanisms identified in fungi, allocated based on the energy management criteria discussed above. At the end of the section, an overview of all retrieved and calculated launch parameters and the associated measurement methods is provided (see Table 1).

**Table 1:** Summary of launch parameters of the identified shooting mechanisms in fungi. For the projectile mass, launch velocity, launch acceleration, launch distance, and launch angle, the measurement technique is coded as following: Standard = measured using a high-speed video camera. **Bold** = calculated by referred authors using measured launch parameters. *Italics* = manual measurement of the parameter (e.g. from photograph stills, without the use of a high-speed video camera). Standard* = estimated by us from data/figure in indicated reference(s); for the power output per unit mass the launch acceleration [m/s^2^] is multiplied with the launch velocity [m/s] and for the work per unit mass the power output [W/kg] is integrated over the launch duration [s]. The launch parameters are indicated as mean (± standard deviation), peak (indicated with “peak” behind the value), or a range (minimum value–maximum value). Per launch parameter, the peak value identified in this review is indicated by a double-lined box (with the exception of the launch angle).

Fungi	Projectile mass [mg]	Launch acceleration [*g*]	Launch velocity [m/s]	Launch distance [m]	Launch angle [°]	Power output [W/kg]	Launch duration [ms]	Work [J/kg]
Osmosis: Water Condensation								
**Elastic Energy Storage—Fracture Release Mechanism**								
Momentum Catapult								
Basidiomycota [32]								
*Aleurodiscus gigasporus* [34]	*0*.*017*	-	0.53	0.0018 peak	-	-^a^	-	-
*Auricularia auricula* [38,40]	*0*.*37·10*^*−6*^	3,302–12,000	1.23 (0.87–1.62)	**45·10**^**−5**^ **± 2·10**^**−5**^	-	2.8·10^4^–1.45·10^5^*	0.01	0.28–1.45*
*Itersonilia perplexans* [35,38]	*1*.*5*·*10*^*−6*^ ^*b*^	~ 25,484	0.67^c^	1·**10**^−3^	75*	1.64·10^5^*	< 0.01*	< 1.64*
*Hyphodontia latitans* [34]	*0*.*6*·*10*^*−9*^	-	1.05	4·10^−6^ peak	-	-^a^	-	-
*Gymnosporangium juniper—virginianae* [40]	*0*.*003*	-	1.11 (0.66–1.35)	**13·10**^**−4**^ **± 6·10**^**−5**^	-	-	-	-
Osmosis: Water Absorption								
**Elastic Energy Storage—Fracture Release Mechanism**								
Fluid Pressure Catapult [56]	1·10^−9^–2·10^−3^							
Ascomycota								
*Ascobolus immersus* [10,41,56]	*0*.*001*	183,486 peak	14^d^ (5–18)	*0*.*3 peak*	-	3.24·10^7^ peak*	< 0.06*	< 1,944*
*Gibberella zeae* [10,43,50]	*0*.*2*·*10*^*−6*^	**870,000**	**34.5**	*2*.*8*·*10*^*−3*^*–8*.*5*·*10*^*−3*^	-	2.94·10^8^*	-	-
*Neurospora tetrasperma* [45]	-	-	1.24 (1.80 peak*)	-	60*	-	-	-
*Podospora anserina* [41]	-	152,905 peak	21^d^ (10–25)	*0*.*2 peak*	-	3.74·10^7^ peak*	< 0.096*	<3,590*
*Sclerotinia sclerotiorum* [46]	-	-	8.4	0.01	90*	-	-	-
*Glomerobolus gelineus* [52]	-	-	-	*0*.*33 peak*	-	-	-	-
Zygomycota								
*Pilobolus*								
*Pilobolus kleinii* [8,10,41,50,53–55]	-	21,407 peak	9^d^ (2–16)	*2*.*5 peak*	70–90*	3.36·10^6^ peak*	0.01–0.03	112.2 peak*
*Basidiobolus*								
*Basidiobolus ranarum* [41,56]	-	152,905 peak	4^d^ (2–9)	*0*.*02 peak*	-	1.35·10^7^ peak*	< 0.024*	< 324*
**Elastic Energy Storage—Eversion Release Mechanism**								
Osmotic-powered Eversion Catapult								
Basidiomycota								
*Sphaerobolus* [9,10,59]	-	-	**10 peak**	*6 peak*	90*	- ^e^	-	-
Osmosis: Water Evaporation								
**Elastic Energy Storage—Cavitation Release Mechanism**								
Cavitation Coiling Catapult								
Ascomycota								
*Deightoniella torulosa* [60]	-	-	-	-	-	-	-	-
*Zygophiala jamaicensis* [61]	-	-	-	-	-	-	-	-
*Curvularia*								
*Curvularia lunata* [61]	-	-	-	-	-	-	-	-
*Curvularia geniculate* [61]	-	-	-	-	-	-	-	-
*Memnoniella subsimplex* [61]	-	-	-	-	-	-	-	-
*Corynespora cassiicola* [61]	-	-	-	-	-	-	-	-
*Alternaria tenuis* [61]	-	-	-	-	-	-	-	-
Basidiomycota	-	-	-	-	-	-	-	-

^a^ Additional information *Aleurodiscus gigasporus* and *Hyphodontia latitans*:

- *A*. *gigasporus*: available surface tension energy 2.9·10^−11^ J, energy to break connection 1.6·10^−14^ J, proportion of total energy consumed in fracture 0.1%, kinetic energy of launch 2.7·10^−12^ J (9.3%).

- *H*. *latitans*: available surface tension energy 2.6·10^−14^ J, energy to break connection 1.6·10^−14^ J, proportion of total energy consumed in fracture 61.5%, kinetic energy of launch 3.4·10^−16^ J (3.4%).

^b^ spore mass 8.4·10^−13^ kg [35]

^c^ Total momentum of drop and spore of approximately 2.4·10^−12^ kg·m/s [35]

^d^ Median value [41]

^e^ The power required for gleba discharge is approximately 0.1 W [59].

### Osmosis: Water condensation

#### Elastic Energy Storage in Sterigma—Fracture Release Mechanism: Momentum Catapult

**Fig 2:**
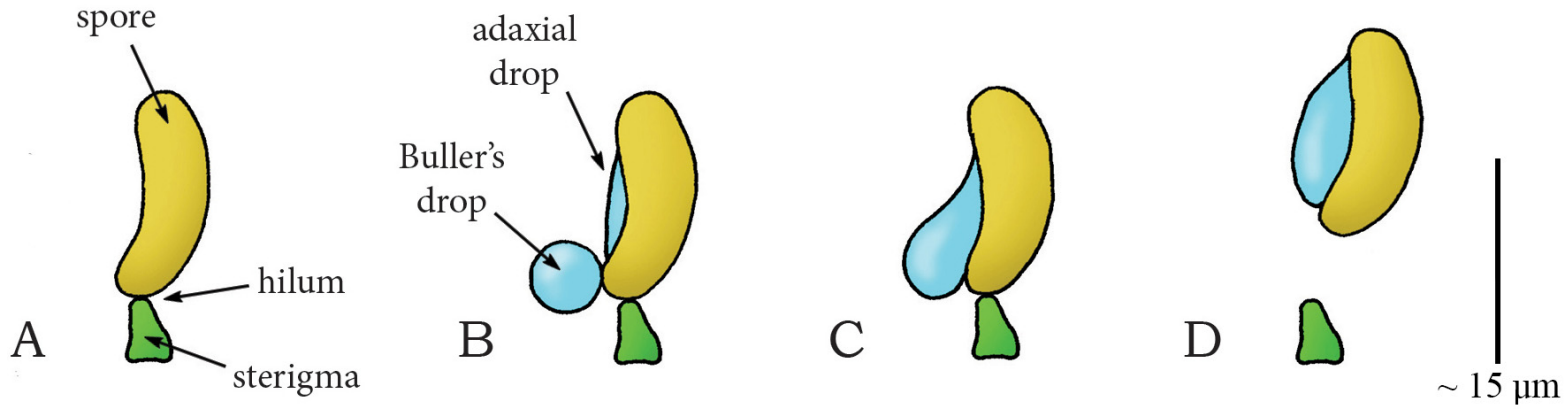
Momentum catapult mechanism in the phylum Basdiomycota (species *Auricularia auricular*). (A) The sporogenous cell of Basdiomycota before discharge with the spore attached to the sterigma at the hilum. (B) By secreting osmolytes, Buller’s drop and the adaxial drop grow on the surface of the spore. (C) When Buller’s drop reaches a critical size, the two drops coalesce, generating a compression force on the sterigma. (D) A rapid shift of the joint center of mass of the spore and the coalesced drops puts tension on the hilum. When a critical tensile stress is reached, the hilum breaks and the spore (together with the coalesced drops) is discharged. Drawings based on schematic drawings in [34]. Scale bar 15 micrometer [μm].

In Basidiomycota, a phylum of fungi including many edible mushrooms, most spores are actively dispersed by a momentum catapult: a shooting mechanism characterized by the coalescence of two water droplets (the so-called Buller’s drop and adaxial drop [32]) on the spore surface that generate the energy needed to discharge the spore(s) by momentum transfer [19,33–37]. The sporogenous cell of the concerning species typically consists of cup-shaped reproductive units called basidiospores or ballistospores (mass without the droplets 8.4·10^−7^ milligram [mg] and with droplets 1.5·10^−6^ mg in *Itersonilia perplexans* [35]), connected to a stalk (sterigma) by the hilum (Fig 2A). Noblin *et al*. [19] describe the shooting mechanism of Basidiomycota as a four-stage process: (1) The nearly spherical Buller’s drop and the hemispherical adaxial drop grow due to the secretion of osmolytes onto the spore surface, lowering the center of mass of the spore (Fig 2A and 2B). (2) Buller’s drop and the adaxial drop reach a critical size, contact each other, and begin to coalesce, generating a compression force on the stalk and a counterforce on the spore (Fig 2B and 2C). (3) The two drops continue to coalesce, leading to momentum transfer from the merged drop to the spore (total momentum of drop and spore of approximately 2.4·10^−12^ kilogram meter per second [kg·m/s] [35]), with the stalk now under a tension force (Fig 2C). (4) The hilum breaks under the tension created by the momentum transfer and the braking of the drop at the spore’s tip [38], releasing the spore (Fig 2D). The variation in the size of the spores and Buller’s drops produces a range of launch accelerations from 3,302 to 25,484*g* [35,38–40], launch velocities from 0.1 to 1.8 m/s [34,38,40], and launch distances from a few thousand of a millimeter [mm] in the smallest spores to a few millimeters in the larger spores [32,34,35,38,40]. Given their small size and mass, spores operate in a low Reynolds number (i.e. a dimensionless quantity that quantifies the relative effect of inertial and viscous drag forces) regime, where friction drag is relatively high. The spores are, therefore, strongly decelerated after release from the sterigma and reach relatively small release distances in spite of the high accelerations. The larger the spore, the least it is affected by drag. It is hypothesized that the species with the shorter ranges (<0.1 mm) propel their spores from fertile tissues, whereas species that discharge their spores over larger distances (≥0.5 mm) liberate them directly into the airstream, thereby (slightly) increasing their probability of escaping the laminar boundary layer [40].

### Osmosis: Water absorption

#### Elastic Energy Storage in Cell Wall—Fracture Release Mechanism: Fluid Pressure Catapult

The fluid pressure catapults, or squirt gun mechanisms, are most common in the largest fungal phylum Ascomycota [10,41–51], including lichenized species (i.e. composite organisms that arise from algae or cyanobacteria and live among the filaments of a fungus in a symbiotic relationship), but have also evolved among the Zygomycota [41].

**Fig 3:**
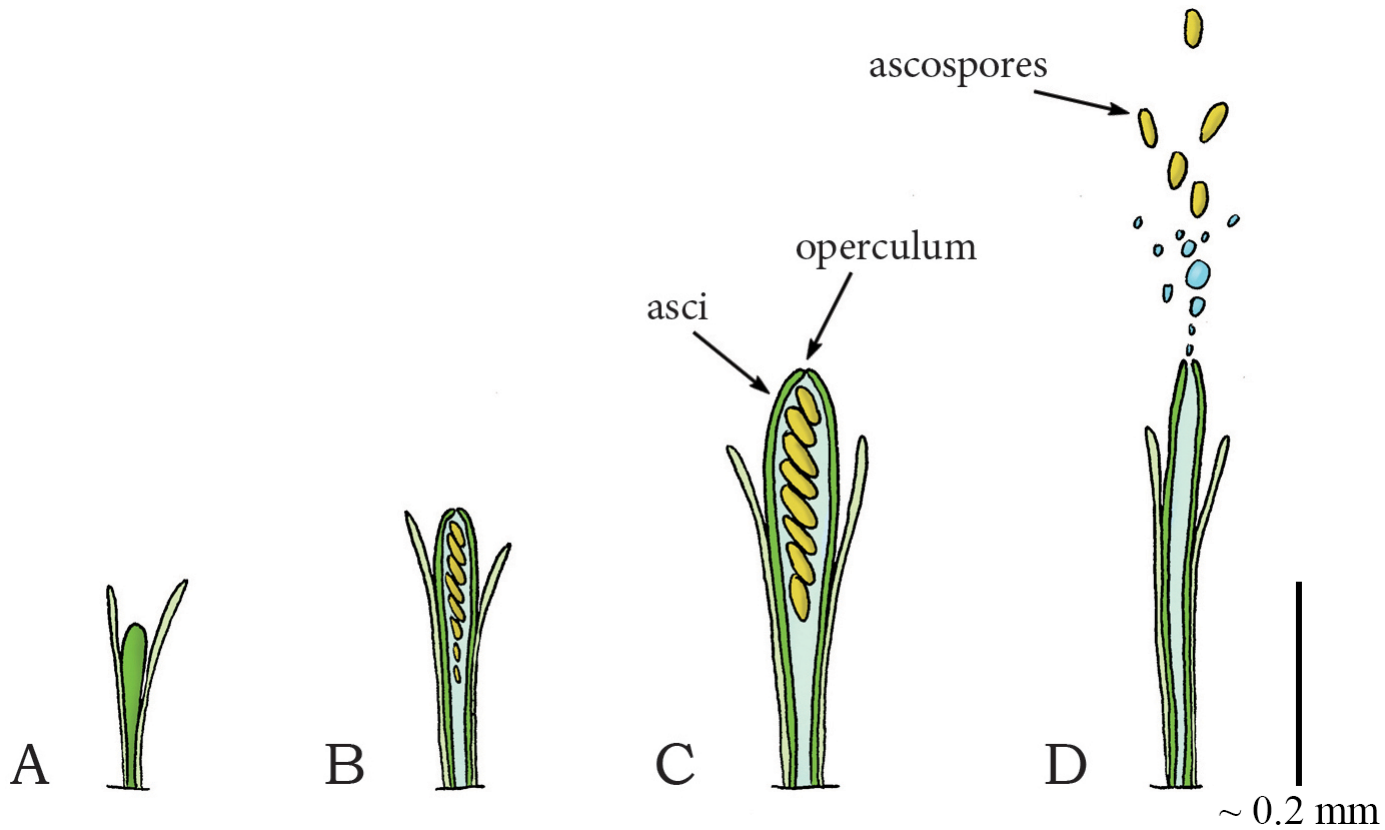
Fluid pressure catapult mechanism in the phylum Ascomycota (species *Ascobolus immersus*). (A) Early stage of ascus development in Ascomycota. (B) Developed ascus containing the ascospores. (C) Osmotic water absorption increases turgor pressure and drives the expansion of the ascus. (D) When a critical pressure is reached (range: 0.30–1.54 MPa), the operculum breaks open, allowing contraction of the expanded wall, which drives the discharge of the ascospores together with the cell sap from the ascus. Drawings based on schematic drawings in [50]. Scale bar 0.2 mm (200 μm).

The defining feature of Ascomycota is their asci: fluid-filled sacs, from which spores (ascospores) are ejected (Fig 3A–3D). Osmolytes inside the ascus lead to inflow of water through the ascus membrane, which increases turgor pressure inside the ascus and causes expansion and stretching of the ascus wall (Fig 3C). After reaching a critical pressure of 0.31–1.54 megapascal [MPa] relative to ambient (in between 3 and 15.4 atmospheres [atm]) [41,43,44,47], the spores are discharged together with the liquid content of the ascus through a pore, slit, or operculum (Fig 3C), located at the tip of the ascus. The highest launch acceleration and velocity are reported for *Gibberella zeae* and are 870,000*g* and 34.5 m/s, respectively [43,50]. The spore and fluid projection is powered by the release of elastic energy from the contracting wall of the ascus. The liquid in the container is nearly incompressible and can, therefore, store only a negligible amount of elastic energy. Launch distances of approximately 0.3 m have been reported for *Ascobolus immersus* [41] and the “spitting” fungus *Glomerobolus gelineus* [52], which is high-enough to reach the turbulent boundary layer, enabling dispersal by wind. It has been shown that, in order to maximize launch distance, energy losses during ejection and drag are minimized by the shape of the operculum [48] and spores [45], respectively. Furthermore, by synchronizing the ejection of thousands of spores, ascomycetes create a favorable flow of air that carries spores through the laminar boundary layer, around intervening obstacles, and towards the turbulent boundary layer, negating the range constraints imposed by (viscous friction) drag, thereby generating 20 times greater launch distances than that of individually discharged spores [46]. Finally, a rotational movement of the spores at 1,200 rotations per minute (comparable to the rotational movement of a bullet after leaving the coiled riffling of a barrel of a gun or cannon [51]) is observed after discharge [51], which can potentially decrease the effect of the wind on the trajectory of the spores. Large launch distances tend to increase the average dispersal distance by wind, which enables the species to reach far-away habitats.

**Fig 4:**
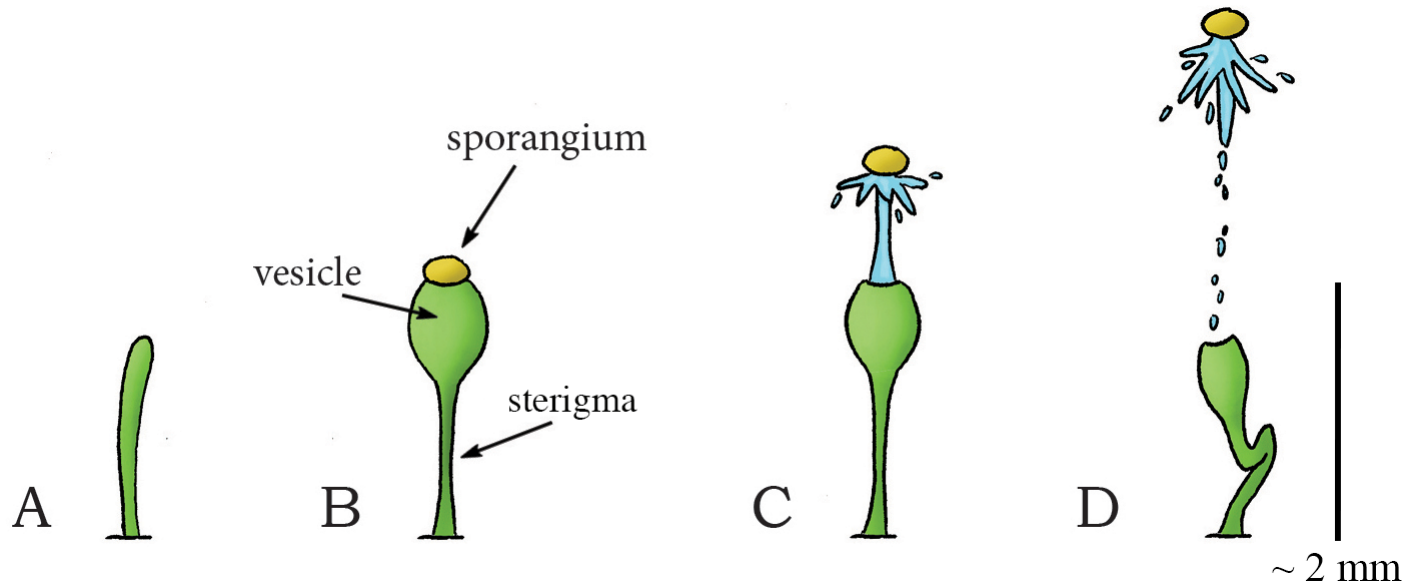
Fluid pressure catapult mechanism in the zygomycete *Pilobolus kleinii*. (A) Early stage of sporangiophore growth in *P*. *kleinii*. (B) Sporangiophore development showing the sterigma (stalk), balloon-like vesicle, and the sporangium at the tip. (C) When a critical pressure (of about 0.55 MPa relative to ambient) is reached, the sporangium breaks free from the sporangiophore and is propelled forward by a cell sap jet that is powered by the contracting vesicle wall. (D) Collapse of the sporangiophore after discharge of the sporangium. Drawings based on high-speed video images in [54]. Scale bar 2 mm.

The fluid pressure catapult is also observed in the genera *Pilobolus* (class: Mucoromycotina, order: Mucorales, family: Pilobolaceae) and *Basidiobolus* (class: Zygomycetes, order: Entomophthorales, family: Basidiobolaceae; recently questions have been raised about the placement of Basidiobolaceae within the Entomophthorales) within the Zygomycota (a phylum of fungi consisting of approximately 1,050 species that are characterized by spherical spores (length = 0.03–0.07 mm [53]) developed for sexual reproduction [41]). *Pilobolus* grows on herbivore dung and is commonly known as the squirt-gun or hat thrower fungus. *Pilobolus* grows spore-producing structures (sporangiophores) that consist of a stalk (sterigma) and a balloon-like vesicle (Fig 4A and 4B). In the common species *Pilobolus kleinii*, a package filled with spores (sporangium; containing between 30.000–90.000 spores [10]) is formed at the tip of the vesicle (Fig 4B). Due to (osmotic) absorption of water, the balloon-like vesicle swells, and the hydrostatic pressure in it increases. When a critical pressure of about 0.55 MPa relative to ambient (about 5.5 atm) [10,41] is reached, the spore package breaks free from the vesicle (in 0.01–0.03 ms [53]) and is propelled by a jet of cell sap with a peak acceleration up to 21,407*g* and a peak launch velocity of 16 m/s (mean: 9 m/s), resulting in a launch distance of 2.5 m for launch angles of 70–90° to the horizontal (estimated from figures in [54]) (Fig 4C and 4D) [10,41,50,55]. Again, the spore and fluid projection is powered by the release of elastic energy from the contracting wall of the vesicle, which is converted into kinetic energy of the ejected spores and some deformation of the stalk of the sporangiophore. The launch distance is larger than in the Ascomycota because the spore package remains intact, resulting in an overall larger mass of the projectile and thus a lower influence of viscous drag (see Eq 1), as the decelerating drag force is (approximately) proportional to the square of the spore radius (*r*^2^), while spore inertia is proportional to its mass and thus to *r*^3^ for a spherical object [40].

Fdrag=12ρAv2Cd, (1)

with *ρ* = density of the fluid [kg·m^3^], *A* = cross-sectional area (πr^2^ for spherical objects) [m^2^], *v* = velocity of the projectile [m/s], and *C*_*d*_ = drag coefficient (i.e. a dimensionless quantity that quantifies the drag of an object in a fluid environment).

*Basidiobolus* is a genus of microscopic fungi that inhabit the guts of small animals and is known to cause rare infections in the host species, including humans [41]. The spore-producing structure of *Basidiobolus ranarum*, for example, is similar to that of *Pilobolus*, with the major difference that *B*. *ranarum* discharges a single spore (called conidium). The wall of the spore-bearing structure ruptures around its circumference with an estimated internal pressure of 0.01–0.72 MPa relative to ambient, and the spore is discharged with a peak launch acceleration of 152,905*g* and a peak launch velocity of 9 m/s (mean: 4 m/s), resulting in a peak launch distance of 0.02 m (with an theoretical maximum of 0.05 m using Stokes drag) [41]. The difference in launch distance between *Pilolobus* and *Basidiobolus* is a physical consequence of the difference of size and thus effect of the viscous drag on the spores (see also Eq 1). This is substantiated in a study of Fischer *et al*. [56], in which the Reynolds number for the launch of *Pilobolus* and *Basidiobolus* were calculated as 167 and 10, respectively, indicating a higher effect of viscous drag in *Basidiobolus*.

#### Elastic Energy Storage in Cell Wall—Eversion Release Mechanism: Osmotic-Powered Eversion Catapult

**Fig 5:**
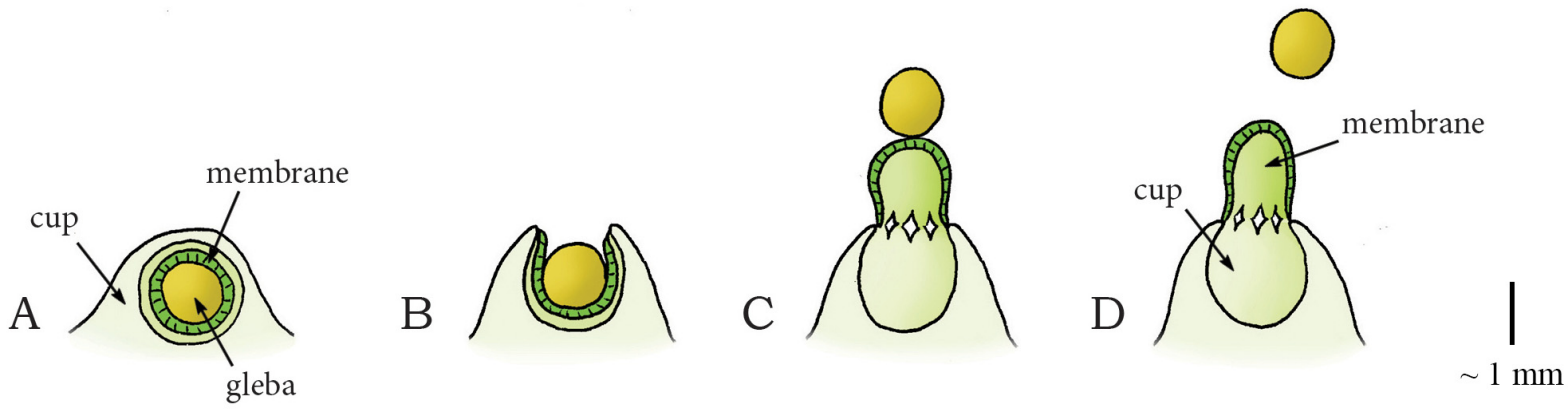
Osmotic-powered eversion catapult mechanism in the genus *Sphaerobolus*. (A) Immature fruiting body of *Sphaerobolus*. (B) The developed fruiting body with the exposed gleba (Ø1 mm, contains the spores) that is supported by an elastic membrane within a firm outer case of the cup. (C–D) When a critical pressure is reached inside the cells of the elastic membrane, the membrane everts rapidly, discharging the gleba from the cup. Scale bar 1 mm.

Another type of active spore dispersal in the phylum Basidiomycota can be found in the genus *Sphaerobolus* (class: Agaricomycetes, order: Gaestrales, family: Geastraceae). *Sphaerobolus* is known as the cannonball fungus or artillery fungus, and is usually found on dung, decaying wood, or vegetative litter [57]. The generic name is deducted from the Greek words sphear, meaning sphere, and obulus, meaning throw, as the fungus discharges a spore-filled round package called gleba (diameter [Ø] of 1 mm). Discharging is led by the osmotic-powered eversion of a membrane that surrounds the package [10], similar to the kids toy “jumping poppers”. The immature base of *Sphaerobolus* is a closed sphere (Fig 5A). At maturity, this sphere splits radially from its apex, forming a toothed (star-shaped) cup that envelops the spore-filled package (Fig 5B) [10]. The cup consists of a firm outer case and an elastic inner membrane (peridium). By solubilization of glycogen and subsequent absorption of water, the turgor pressure within the radially oriented cells of the elastic inner membrane increases [10,58]. As the inner membrane has a concave form, the exposed ends of the radially orientated cells are more compressed than their bases, resulting in strain within the cell walls. The sudden eversion of the membrane relieves the strain and discharges the spore package (approximately vertically) with an estimated peak launch velocity of 10 m/s, reaching up to 6 m from its base (Fig 5C and 5D) [9,59]. The large launch distance (partly) compensates for a low efficacy of wind dispersal, as the gleba is most likely too heavy to be swept away over large distances by the wind.

### Osmosis: Water evaporation

#### Elastic Energy Storage in Cell Wall—Cavitation Release Mechanism: Cavitation Coiling Catapult

Cavitation-based spore discharge is observed in fungi imperfecti (i.e. fungi that do not fit into the commonly established taxonomic classifications). The fungi imperfecti represent asexually reproducing genera in the phyla Ascomycota and Basidiomycota. Cavitation-based spore discharge was first observed in *Deightoniella torulosa* (incertae sedis, phylum: Ascomycota, class: Dothideomycetes, order: Capnodiales, family: Mycosphaerellaceae), a pathogen causing banana fruit-spot [60]. This fungus grows its spores on stalks called conidiophores. When dehydration causes the cell membranes to shrink, the cell walls start caving inwards, which increases the tension within these walls and slowly deforms the structure. Continuous negative pressure that exceeds the tensile strength of the cytoplasm causes the cytoplasm to fracture, resulting in the appearance of a gas bubble (or cavitation bubble) that releases the wall tension and causes the walls to rapidly return to their original shape (similar to a coiling motion), catapulting the spores out of the conidiophore [60].

**Fig 6:**
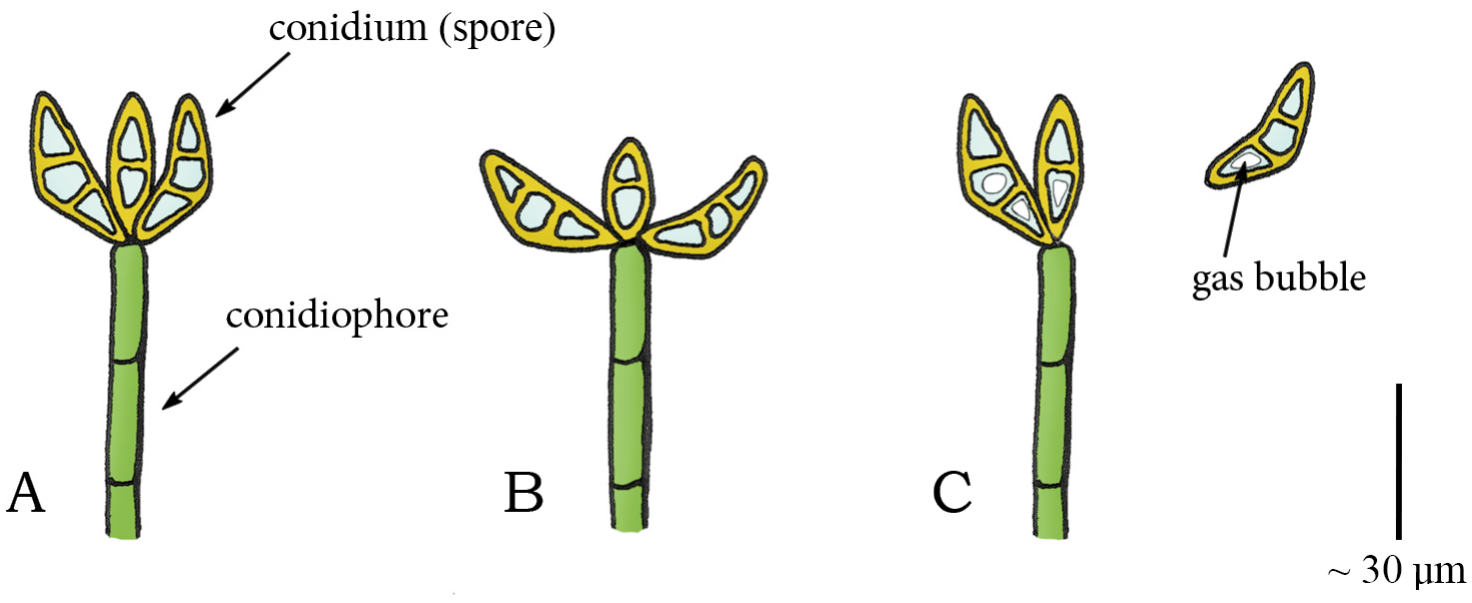
Cavitation catapult mechanism in *Curvularia*. (A) Conidiophore in *Curvularia* with a cluster of boat-shaped conidia (spores) at its apex. (B) Outward movement of the conidia caused by drying. (C) When a critical negative pressure (relative to ambient) is reached, the sudden appearance of a gas bubble in the conidia releases the stored elastic energy and causes a rapid return movement of the conidia to their original shape, which disrupts the connection with the conidiophore and launches the conidia. Drawings based on schematic drawings in [61]. Scale bar 30 μm.

Species of the genus *Curvularia* (phylum: Ascomycota, class: Euascomycetes, order: Pleosporales, family: Pleosporaceae) are pathogens of plants and soil that are found in tropical regions. In at least two species, *C*. *lunata* and *C*. *geniculate*, spore discharge is also triggered by cavitation. In these species, the young conidiophore bears a cluster of boat-shaped (asexual) spores (conidia) at its apex (Fig 6A) [61]. During drying, the spores slowly move either inwards, outwards, or to the side, with respect to the attachment to the conidiophore (Fig 6B). When a critical negative pressure is reached in a spore, cavitation bubbles appear, resulting in a rapid return movement of the spore to their original shape which disrupts the connection with the conidiophore and launches the spore (Fig 6C) [61].

**Fig 7:**
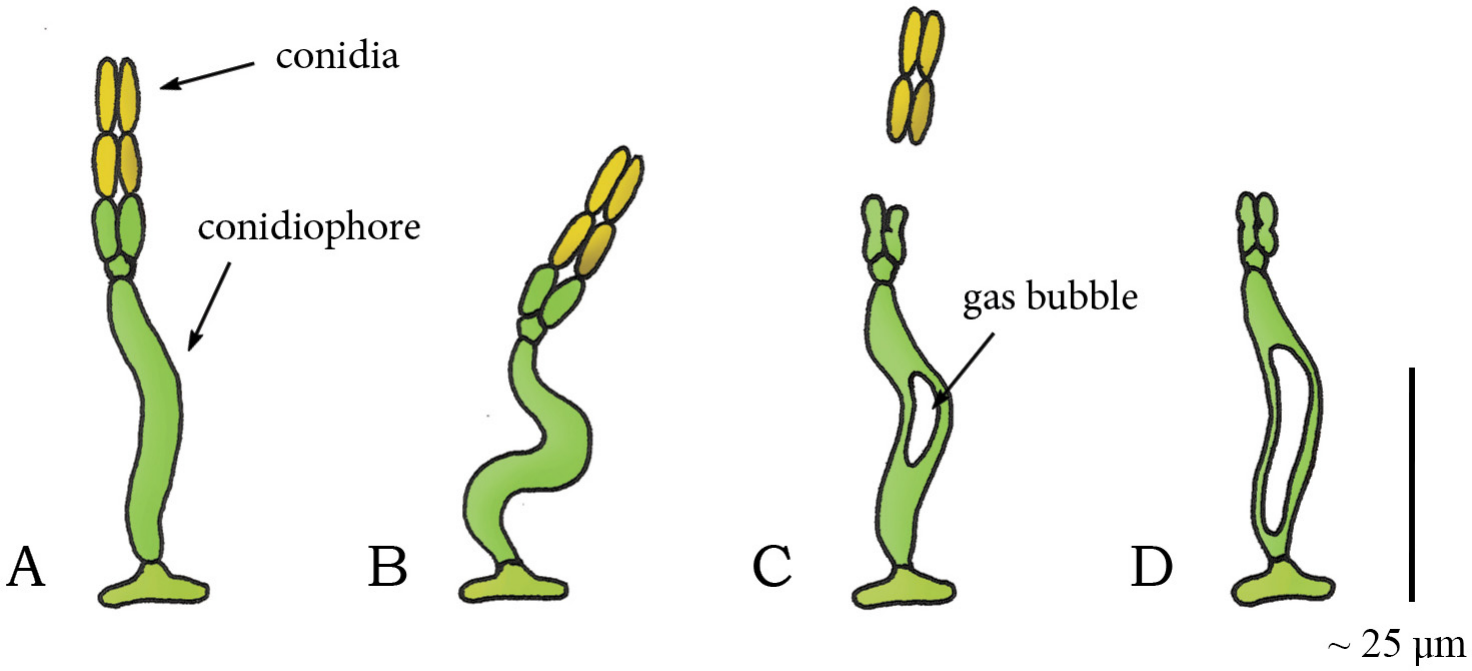
Cavitation catapult mechanism in *Zygophalia jamaicensis*. (A) Conidiophore of *Z*. *jamaicensis* with two divergent conidia at its apex. (B) S-shaped compression of the conidiophore by drying. (C) When a critical negative pressure (compared to ambient) is reached, the sudden appearance of the gas bubble in the conidiophore releases the stored elastic energy in the cell walls and discharges the conidia from the sporogenous cells. (D) Conidiophore after discharge. Drawings based on schematic drawings in [61]. Scale bar 25 μm.

A cavitation coiling catapult mechanism is also observed in the species *Zygophiala jamaicensis* (phylum: Ascomycota, class: Dothideomycetes, order: Microthyriales, family: Schizothyriaceae). This pathogen grows on banana leafs and consists of a conidiophore with two sporogenous cells and conidia at the apex of the conidiophore (Fig 7A). Drying of the conidiophore causes it to deform into an S-shape (Fig 7B) [61]. At the sudden appearance of a gas bubble, the conidiophore springs back to its former shape, discharging the conidia from the sporogenous cells (Fig 7C and 7D) [61].

**Fig 8:**
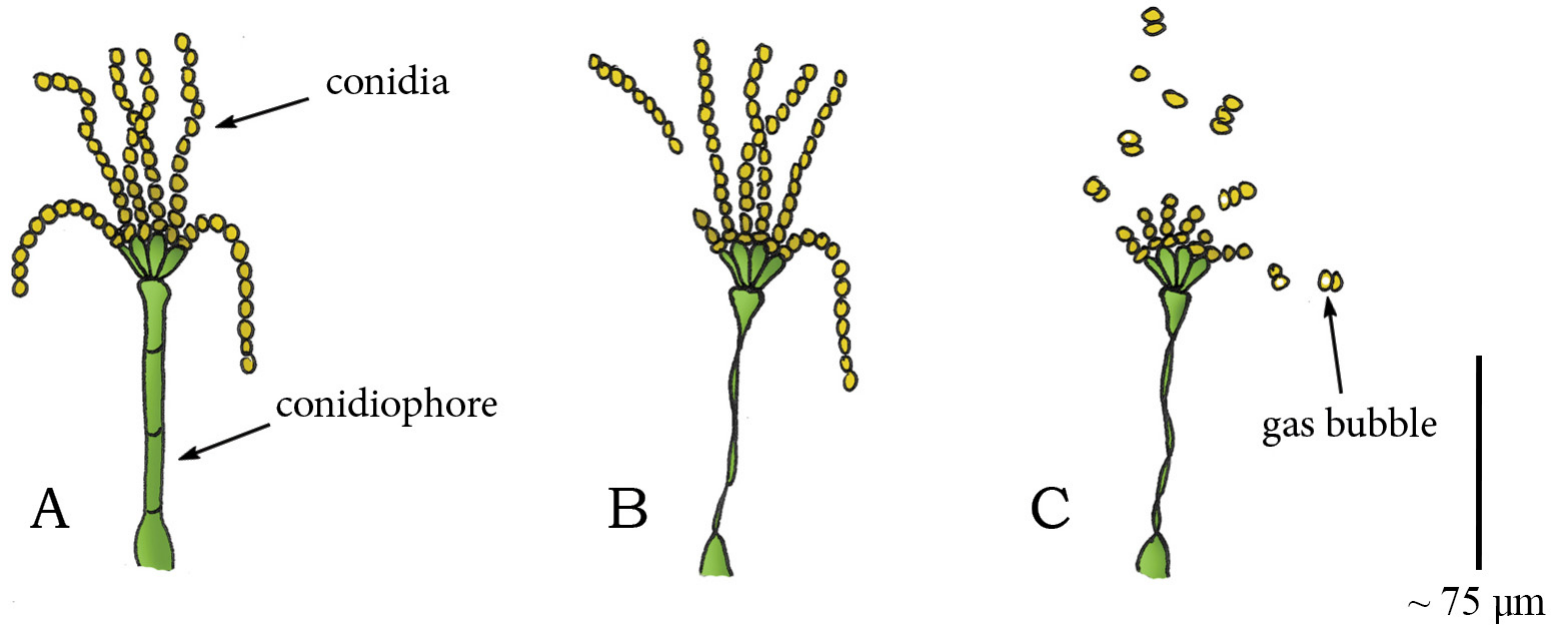
Cavitation catapult mechanism in *Memnoniella subsimplex*. (A) Conidiophore of *M*. *subsimplex* with conidia in a chain-like fashion at its apex. (B) Twisting of the conidiophore during drying discharges loosely connected conidia. (C) In [61] it is suggested that a critical “negative” pressure (compared to ambient) causes the sudden appearance of gas bubbles in the conidia that releases the tension in the cell walls of the conidia, resulting in a rapid return motion, and the subsequent discharge of the conidia. Drawings based on schematic drawings in [61]. Scale bar 75 μm.

Meredith *et al*. [61] suggested that the cavitation coiling catapult mechanism also holds for *Memnoniella subsimplex* (phylum: Ascomycota, class: Sordariomycetes, order: Hypocreales, family: Stachybotriaceae), a common invader of decaying banana leafs. This fungus consists of erect, straight conidiophores [61]. The (asexual) spores are formed in a chain-like fashion, with each chain containing as many as 25 spores (Fig 8A). Drying causes the conidiophore to twist about its longitudinal axis and rapidly rotate through nearly 360° (Fig 8B). Although the twisting motion of the conidiophore greatly assists the detachment of loosely connected spores [61], Meredith [61] speculates instead that the energy for discharge is related to the appearance of gas bubbles in the conidia (Fig 8C). A similar twisting motion and appearance of gas bubbles is observed in *Corynespora cassiicola* (phylum: Ascomycota, class: Dothideomycetes, order: Pleosporales, family: Corynesporascaceae) and in *Alternaria tenuis* (phylum: Ascomycota, class: Dothideomycetes, order: Pleosporales, family: Pleosporaceae), both containing conidia, borne singly or in chains of 2–6 at the apex of the conidiophore [61].

Unfortunately little is known about the launch acceleration, velocity, and distance of cavitation-based spore discharge in fungi imperfecti. Furthermore, the working principles of the cavitation-based spore discharge in many fungal species, such as those in *M*. *subsimplex* [58], are still to be determined.

## Plants

**Fig 9:**
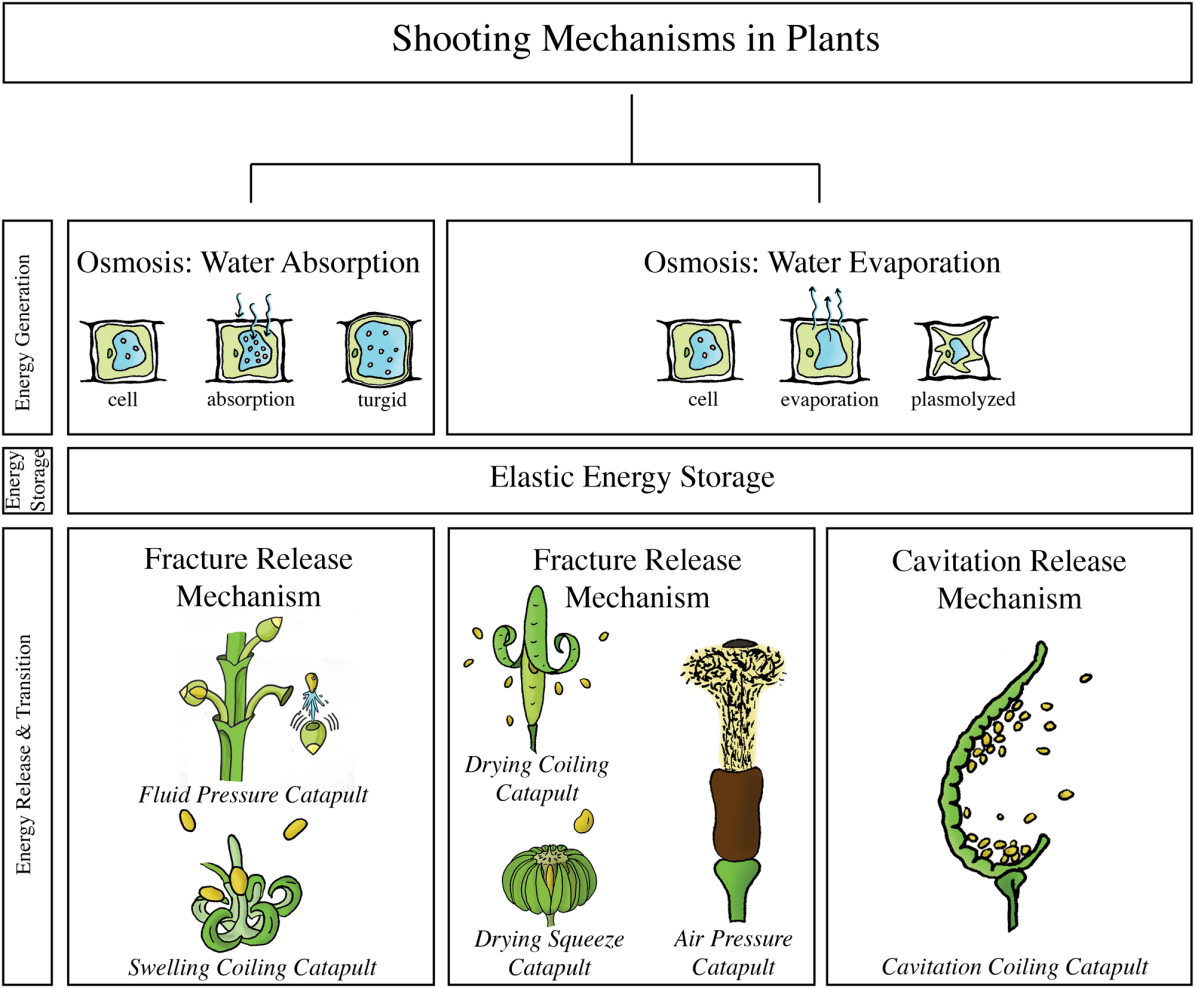
The structural categorization of the identified shooting mechanisms in plants, allocated based on the energy management criteria discussed above. **Fluid pressure catapult**: observed in the genus *Arceuthobium*. **Swelling coiling catapult**: observed in the genus *Impatiens*, *Cornus canadensis*, and *Morus alba*, schematic illustration of *Impatiens capensis*, **Drying coiling catapult**: observed in the genus *Cardamine* and the family Fabaceae, schematic illustration of *Cardamine parviflora*. **Drying squeeze catapult**: observed in the family Euphorbiaceae, the Rutaceae family, the genus *Illicium*, the species *Oxalis acetosella*, and the *Viola* family, schematic illustration of *Hura crepitans*. **Air pressure catapult**: observed in the genus *Sphagnum*. **Cavitation coiling catapult**: observed in the family Polypdiaceae and genus *Selaginella*, schematic illustration of *Polypodium aureum*. In contrast to the situation in fungi, no shooting mechanisms were identified in the water condensation category.

Fig 9 illustrates the shooting mechanisms identified in plants, allocated based on the energy management criteria discussed above. An overview of all retrieved launch parameters and the associated measurement methods is provided in Table 2.

**Table 2:** Summary of launch parameters of the identified shooting mechanisms in plants. For the projectile mass, launch velocity, launch acceleration, launch distance, and launch angle, the measurement technique is coded as following: Standard = measured using a high-speed video camera.**Bold** = calculated by referred authors using measured launch parameters. *Italics* = manual measurement of the parameter (e.g. from photograph stills, without the use of a high-speed video camera). Underlined = calculated by referred authors using a mathematical model of the shooting mechanism. Standard* = estimated by us from data/figure in indicated reference(s); for the power output per unit mass the launch acceleration [m/s^2^] is multiplied with the launch velocity [m/s] and for the work per unit mass the power output [W/kg] is integrated over the launch duration [s]. Bold* = calculated by us using the mean of the parameter ± 3 standard deviations. The launch parameters are indicated as mean (± standard deviation), peak (indicated with “peak” behind the value), or a range (minimum value–maximum value). Per launch parameter, the peak value identified in this review is indicated by a double-lined box (with the exception of the launch angle).

Plants	Projectile mass [mg]	Launch acceleration [*g*]	Launch velocity [m/s]	Launch distance [m]	Launch angle [°]	Power output [W/kg]	Launch duration [ms]	Work [J/kg]
Osmosis: Water Absorption								
**Elastic Energy Storage—Fracture Release Mechanism**								
Fluid Pressure Catapult								
*Arceuthobium* [53,65–68]					-	-	0.1–0.2*	-
*Arceuthobium americanum* [67]	*2*.*0*	-	26.1 ± 0.2	-	-	-	-	-
*Arceuthobium cyanocarpum* [67]	*0*.*9*	-	21.3 ± 0.3	-	-	-	-	-
*Arceuthobium cryptopodum* [67,68]	*2*.*3*	-	25.4 ± 0.3	*14*.*6 peak*	-	-	-	-
*Arceuthobium douglasii* [67]	-	-	22.3 ± 0.4	-	-	-	-	-
*Arceuthobium vaginatum* [65]	-	**~ 4,791**	**~ 13.7**	*4*.*6*	-	-	**~ 0.44**	-
Swelling Coiling Catapult								
*Impatiens*								
*Impatiens capensis* [69]	*10*.*7 ± 0*.*4 (7*.*7–19*.*7)*	-	1.24 ± 0.14 (4.1 peak)	1.75 peak	17.4 ± 5.2	-	4.2 ± 0.4	- ^a^
*Impatiens glandulifera* [70,71]	*20*.*7 (8*.*8–38*.*3)*	-	6.19 (2.57–12.4)	*0–10*	47.8 (-37.1–79.7)	-	-	-
*Cornus canadensis* [72,73]	*0*.*024*	2,446 ± 612	3.1 ± 0.5	0.025 (0.022–0.027)	70–90*	7.3·10^4^ * **(1.9**·**10**^**5**^ peak*)	0.5	36.5 (95 peak)*
*Morus alba* [74]	-	2,500 peak^b^	170–237^b^	-	0–180*	2.6·10^5^ (5.81·10^6^ peak*)	≤0.025	≤6.5 (≤145.3 peak*)
Osmosis: Water Evaporation								
**Elastic Energy Storage—Fracture Release Mechanism**								
Drying Coiling Catapult								
Brassicaceae								
*Cardamine*								
*Cardamine parviflora* [75,76]	*0*.*15 ± 0*.*09*	-	6.29 ± 2.73	0.94 ±0.46	52.2 ± 23.9	1.9·10^4^ * **(2.64**·**10**^**5**^ peak*)^c^	4.7 ± 1.3	89.3 ± 40.5
Fabaceae								
*Cytisus multiflorus* [77]	-	-	-	4 peak	-	-	-	-
*Tetraberlinia moreliana* [78]	*2*,*530*	-	**37.1**	*60 peak*	*17*.*3 ± 11*	-	-	-
Acanthaceae								
*Ruellia simplex* [81]	1.78	-	-	2–3	40	-	-	-
Geraniaceae								
*Geranium* [62]								
*Geranium carolinianum*	*3*.*5 ± 0*.*3*	-	-	*3*.*29 ± 0*.*7*	*45*.*5 ± 3*.*1*	-	-	-
*Geranium maculatum*	*6 ± 0*.*9*	-	-	*3*.*02 ± 0*.*76*	*47*.*3± 10*.*0*	-	-	-
*Geranium molle*	*1*.*1 ± 0*.*1*	-	-	*1*.*79 ± 0*.*43*	*61*.*2 ± 2*.*1*	-	-	-
*Erodium*								
*Erodium cicutarium* [82]	*5 ± 1*	-	4 ± 2^d^	0.51 ± 0.08	40 ± 30	-	-	-^e^
Drying Squeeze Catapult								
Euphorbiaceae								
*Hura crepitans* [8,79,84]	*1*,*020 (700–1*,*430)*	-	43 (14–70)	*30 (45 peak)*	*34*.*2 (20–48)*	-	0.01–0.035*	-
*Mercurialis annua* [83]	*0*.*7–3*.*6*	-	-	*0*.*41 ± 0*.*31 (1*.*3 peak)*	-	-	-	-
Rutaceae								
*Metrodorea nigra* [85]	-	-	-	-	-	-	-	-
*Illicium*								
*Illicium floridanum* [86]	*45 ± 4 (50 peak)*	-	-	*2*.*5 ± 1*.*4 (5*.*8 peak)*	*40–60*	-	-	-
*Oxalis acetosella* [87]	*0*.*9 (0*.*3–1*.*5)*	-	-	*2 peak*	-	-	-	-
*Viola* [62,88]								
*Viola blanda*	-	-	-	*1 (0*.*1–3*.*8)*	-	-	-	-
*Viola curcullata*	-	-	-	*1*.*5 (0*.*1–2*.*1)*	-	-	-	-
*Viola eriocarpa*	*6 ± 0*.*9*	-	-	*1*.*2 (0*.*2–5*.*4)*	*67*.*5 ± 14*.*5*	-	-	-
*Viola papilionacea*	-	-	-	*2*.*1 (0*.*05–4*.*8)*	-	-	-	-
*Viola pedata*	-	-	-	*1*.*4 (0*.*25–5*.*1)*	-	-	-	-
*Viola rostrata*	-	-	-	*1*.*2(0*.*1–4*.*2)*	-	-	-	-
*Viola striata*	*1*.*1* ± *0*.*2*	-	-	*1*.*5 (0*.*4–3)*	-	-	-	-
Air Pressure Catapult								
*Sphagnum* [1,33,89,90]	-	- (36,697 peak)	16 (30 peak)	0.15 (0.1–0.2)	80–90*	-(1.08·10^7^ peak*)	0.01	- (108 peak*)^f^
**Elastic Energy Storage—Cavitation Release Mechanism**								
Cavitation Coiling Catapult								
Polypodiaceae (common ferns) [94,95]								
*Polypodium aureum* [94,95]	-	**~ 100,000**	10	*0*.*01–0*.*02*	-	9.81·10^6^ *	≤ 0.01	≤ 98.1*
*Adiantum peruvianum* [93]	-	6,320 at 3.1 m/s	2.4 ± 1 (5.0 peak)	0.057 peak*	-	1.48·10^5^ (3.09·10^5^ peak)*	0.01*	1.48 (3.09 peak)*
*Selaginella*								
*Selaginella martensii* [96]	-	-	- (0.6)^g^	*0*.*01–0*.*06*^g^	-	-	< 1	-

^a^ Stored elastic energy: 8.87 J (efficiency of 0.5%) [69]

^b^ Average angular velocity: 69,800 rad/s and peak angular acceleration: 5·10^6^ rad/s^2^ [74]

^c^ Mean estimated stored energy: 0.482 ± 0.219 J (21.3 ± 10.3% efficiency) [75]

^d^ Initial angular velocity: 200 ± 100 rad/s [82]

^e^ Estimated released elastic energy: 2.72·10^−3^ J [82]

^f^ Energy stored in the compressed air: 0.27 mJ [1]

^g^ Indicated values for microspore discharge. Macrospore (mass 0.0014 mg) discharge via drying squeeze catapult with a mean launch velocity of 4.5 m/s and a mean launch distance of 0.21 m (peak 0.65 m) [96]. The estimated impulse of one macrospore is 6.3 pN·s [96].

For some of the described species that ballistically disperse their seeds or pollen, a description of the working principle of the shooting mechanism was not found in our literature search, for example, for *Phlox drummondii* (phylum: Tracheophyta, class: Magnoliopsida, order: Ericales, family: Polemoniaceae) [62]. These species will not be discussed, as placement is uncertain.

### Osmosis: Water absorption

#### Elastic Energy Storage in Cell Wall—Fracture Release Mechanism: Fluid Pressure Catapult

**Fig 10:**
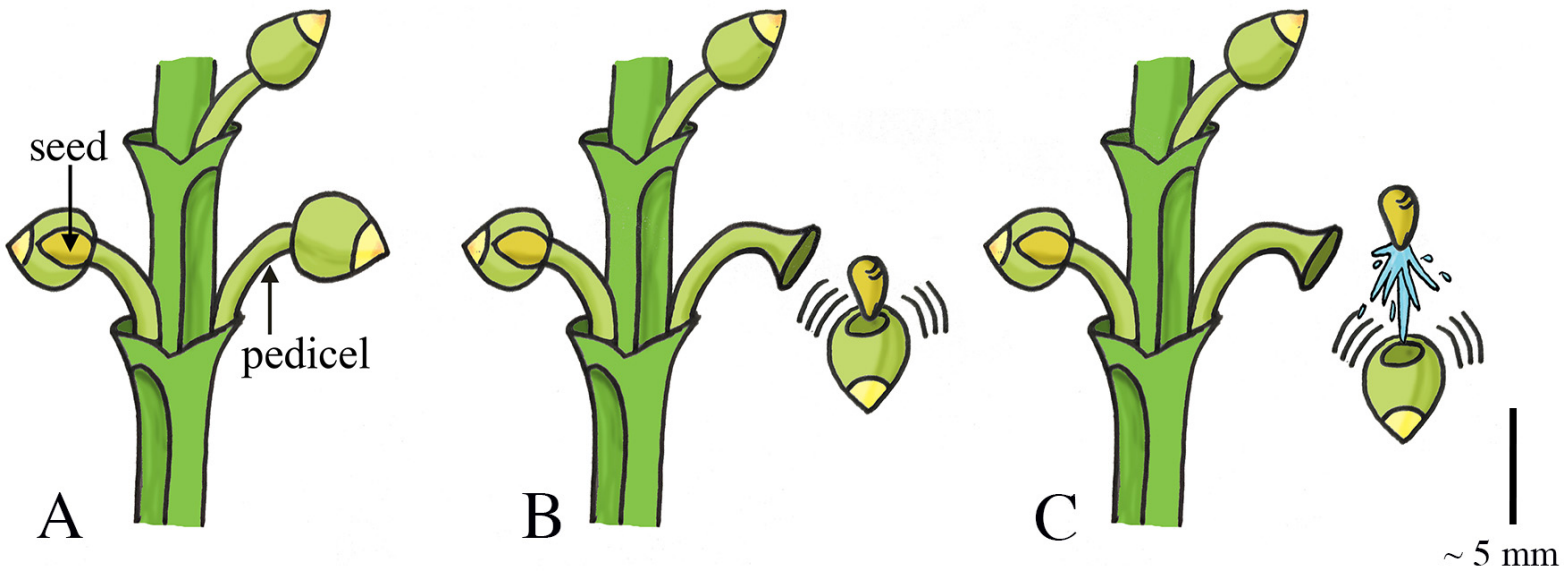
Fluid pressure catapult mechanism in *Arceuthobium*. (A) The ripe fruits of *Arceuthobium* that contains the fusiform-spheric seeds and are attached to short stems (pedicels). (B–C) When a critical pressure is reached, the fruit breaks free from the pedicel and discharges the seed together with the liquid cell content. Scale bar 5 mm. Drawings based on schematic drawings in [65].

*Arceuthobium* (phylum: Tracheophyta, class: Magnoliopsida, order: Santalales, family: Santalaceae (sandalwoods)), commonly known as dwarf mistletoes, is a genus of plants that parasitizes members of Pinaceae and Cupressaceae in Africa, Asia, Europe, Central America, and North America. The ripe fruit of dwarf mistletoes consists of broadly fusiform-spheric seeds attached on short stems (pedicels; Fig 10A) [63,64]. An abscission zone, representing the weakest region of the fruit, develops between the stems and the base of the fruit [65]. Inside the fruit, a layer of viscin tissue surrounds each seed (mass of 2–3 mg [65,66]). During swelling of the fruit, the viscin tissue expands and starts to exert a hydrostatic force on the seeds and a tensile stress in the cell walls. After a critical pressure is reached, the cell walls of the pedicel break at the abscission zone, discharging the seeds (in approximately 4.4·10^**−4**^ s [65]) and liquid cell content with a deducted initial launch acceleration of 4,791g and launch velocity of 13.7 m/s (terminal velocity 7.5 m/s) in *A*. *vaginatum* [65]. Hinds et al. [67] measured the initial mean velocity of the seeds of four dwarf mistletoe species *A*. *cyanocarpum* (seed mass 0.9 mg), *A*. *douglasii*, *A*. *cryptopodum* (seed mass 2.3 mg), and A. americanum (seed mass 2.0 mg) as 21.3, 22.3, 25.4, and 26.1 m/s, respectively (mean of the four species together 24 m/s). The highest measured launch distance is 14.6 m in *A*. *cryptopodum* [68] (Fig 10B and 10C). The data by Hawksworth et al. [65] and Hinds et al. [67] is used by Robinson et al. [66] to compute a computer model to determine the epidemiology of dwarf mistletoes.

#### Elastic Energy Storage in Cell Wall—Fracture Release Mechanism: Swelling Coiling Catapult

**Fig 11:**
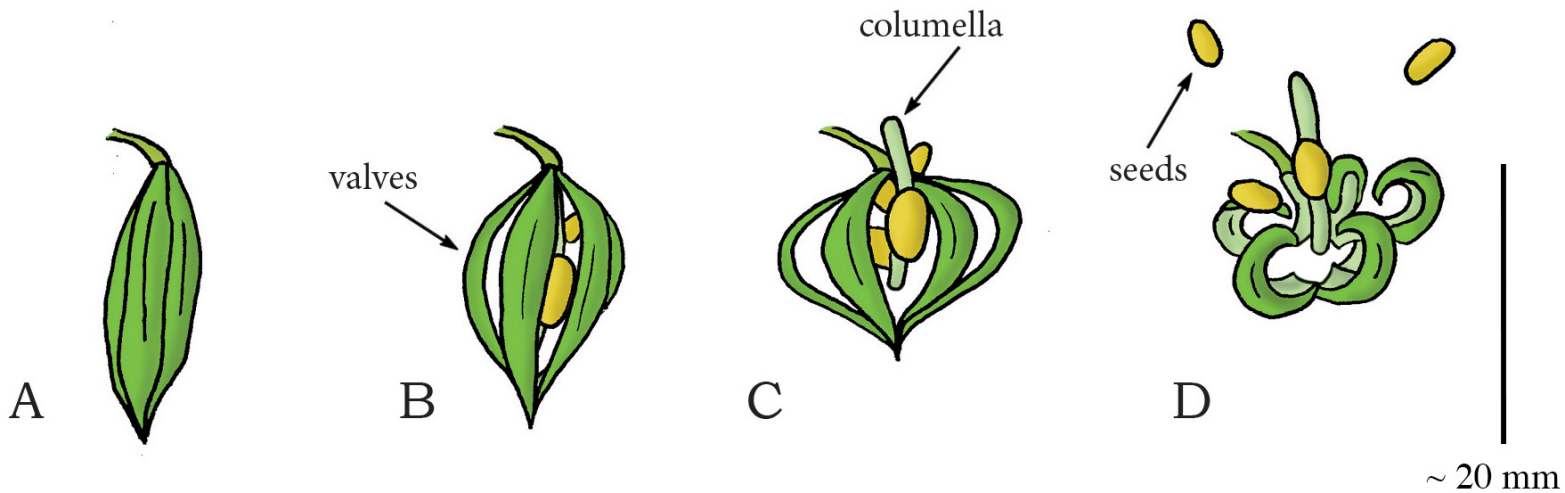
Swelling coiling catapult mechanism in *Impatiens capensis*. (A) The seedpod consisting of five interconnected valves. Elastic energy is stored in the seedpod by the absorption of water in the valves. When a critical pressure is reached, dehiscence of the valves from the columella and subsequent coiling discharges the seeds (A–D). (A) Shows the situation at *t* = 0 ms. Duration from (A) to (D) lasts about 3 to 4 ms. Drawings based on schematic drawings in [69]. Scale bar 20 mm.

The genus *Impatiens* (phylum: Tracheophyta, class: Magnoliopsida, order: Ericales, family: Balsaminaceae), also known as jewelweed and touch-me-not, contains about 850 to 1,000 species of flowering plants found across the Northern Hemisphere and the tropics. In this genus, reproductive units are launched (without fluidic discharge) by a coiling motion of the plant cells. *I*. *capensis* and *I*. *glandulifera* grow capsules that consist of five valves around a central stalk (the columella) (Fig 11A). The valves contain a bilayer structure [69]: an inner cell-layer that shortens and an outer cell-layer that expands by water absorption. Specifically, as the deformation of the adjacent valves is obstructed by their connection to the columella, the inflow of water in the valves tensions the inner cell-layer and compresses the outer cell-layer (Fig 11B and 11C), which in turn results in storage of elastic energy of up to 124 J/kg in the valves of *I*. *patiens* [69]. Release of tension, as a result of dehiscence of the valves from the columella, creates an inward curvature in the valves, shortening the formerly expanded tension-bearing layer and expanding the formerly compressed layer (Fig 11B–11D). When the cracks between the valves reach a critical length, rapid (complete) dehiscence allow the valves to rapidly (3 ms) coil towards their relaxed coiled shape, which transforms the stored elastic energy of 8,870 micro-Joule [μJ] into kinetic energy of 0.2–89 μJ of the seeds in *I*. *capensis* [69] (mean mass 10.7 mg)—an efficiency of approximately 0.5% (the remaining 95% of the energy is used for crack propagation and is dissipated). This results in a peak launch velocity of 4.1 m/s (mean: 1.24 m/s) and a peak seed launch distance of approximately 1.75 m with a mean launch angle of 17.4° to the horizontal in *I*. *capensis* [69]. In *I*. *glandulifera*, the stored elastic energy is transformed into 0.4 mJ kinetic energy of the seeds (an efficiency of approximately 44%) [70], resulting in a peak launch velocity of 12.4 m/s (mean: 6.2 m/s), a peak launch distance of 10 m (mean: 3 m), and a mean launch angle of 47.8° to the horizontal [71].

**Fig 12:**
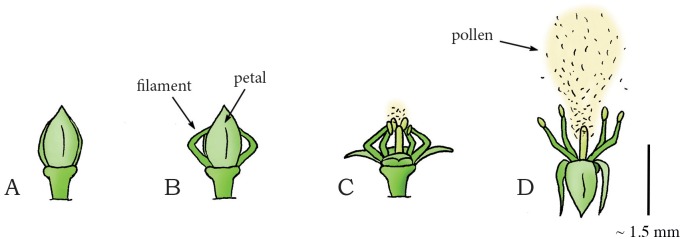
Swelling coiling catapult mechanism in *Cornus canadensis*. (A) Immature flower bud of *C*. *canadensis*. (B) Mature flower bud with filaments emerging from between the petals as the former have grown faster than the latter. (C–D) When a critical pressure is reached, dehiscence of the connection that hold the petals together allow the petals and filaments to unfold rapidly, releasing the stored elastic energy and discharging the pollen into the air. Drawings based on high-speed video images in [73]. Scale bar 1.5 mm.

The species *Cornus canadensis* (phylum: Tracheophyta, class: Magnoliopsida, order: Cornales, family: Cornaceae (dogwoods)), commonly known as bunchberry dogwood, is an herbaceous subshrub with white flowers and red fruits. *C*. *canadensis* has a slightly different coiling catapult mechanism for dispersing pollen than that of *Impatiens*. The flower bud of *C*. *canadensis* contains four interconnected petals and four stamens (Fig 12A and 12B). During flower development, the filaments of the stamens grow faster than the petals. Since the upper ends of the filaments are held in place by the petals, the filaments bend (thereby storing elastic energy) and emerge from between the petals (Fig 12A and 12B). By fracture of the petal connection, the petals rapidly (~ 0.3 ms [72]) separate and flip backwards, allowing the filaments to unfold and accelerate vertically (similar to the way a baseball is accelerated by the sequential deployment of a pitcher’s shoulder, elbow, and wrist [33] and a miniature medieval trebuchet [73]) (Fig 12C). When the peak vertical velocity is reached (~0.5 ms after petal opening [72]), the filaments start accelerating horizontally and separate from each other, releasing the pollen (mass 0.024 mg [72]) with a mean launch acceleration of 2,446*g* and a peak vertical component of the launch velocity of 7.5 m/s [72,73] (Fig 12D). The pollen grains are launched to a height of about 0.027 m with a launch angle of 70–90° to the horizontal (estimated from a figure in [73]) [73].

**Fig 13:**
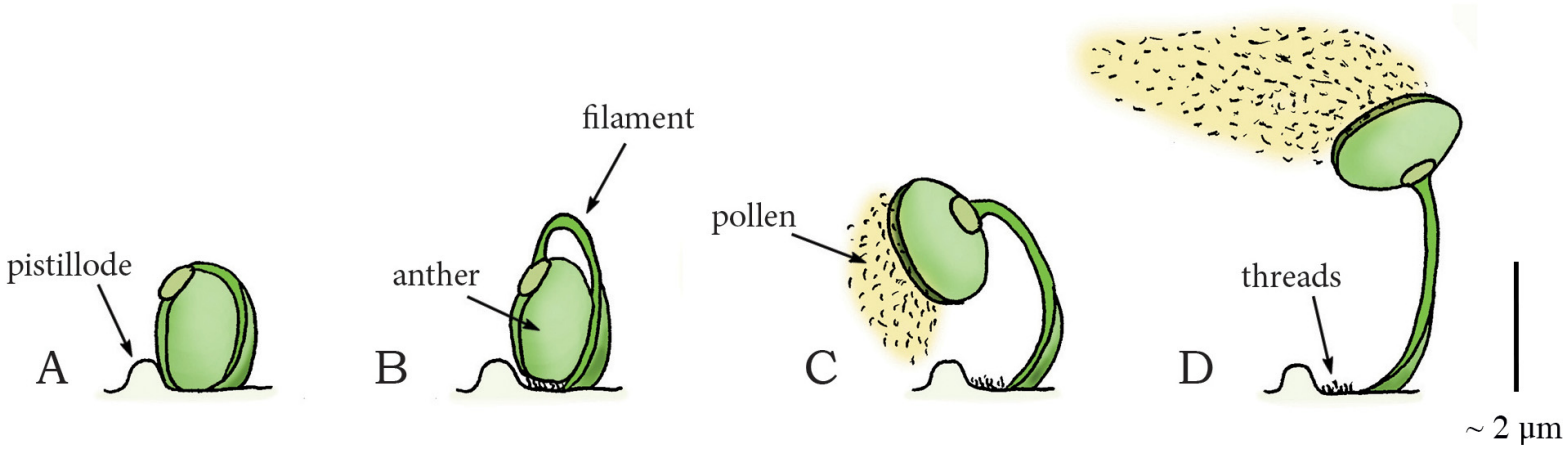
Swelling coiling catapult mechanism in the mulberry *Morus alba*. (A) One of the four filaments with attached anther in the flower bud of *M*. *alba*. (B) The pressurized filament grows and bends, storing elastic energy as deformation is obstructed by the fine thread connections and the pistillode. (C) Slight drying of the anther tears the thread connections. (D) The anther is catapulted in an approximately circular arch driven by the stored elastic energy of the filament while releasing the pollen. Drawings are based on schematic drawings in [74]. Scale bar 2 μm.

Another example of a coiling catapult that aids in pollen dispersal is found in the flower buds of *Morus alba* (phylum: Tracheophyta, class: Magnoliopsida, order: Rosales, family: Moraceae (mulberries)), commonly known as the white mulberry tree. *M*. *alba*, native to northern China, reaches 10 to 20 m in height. The tree has tear-shaped leafs and white fruits and is widely cultivated to feed commercially grown silkworms. The flower bud of this tree contains four stamens. Each stamen consists of a filament with an anther (that contains the pollen) attached at its apex. The anther, in turn, is constrained in movement by the pistillode (i.e. the female ovule-bearing part of the flower bud) and fine threads (Fig 13A and 13B). By water absorption, turgor pressure in the filaments increases and the filaments bend, storing elastic energy [74]. A drop in relative humidity of the environment leads to slight drying of the anther, which in turn causes the pollen-laden anther to pull away from the base of the filament, tearing the fine threads by which the anther was closed and held in place (Fig 13A and 13B). When the anther subsequently slides off the pistillode, the stored elastic energy in the filament is released, catapulting the anther in an approximately circular path with a peak angular launch acceleration of 5,000,000 radian per second squared [rad/s^2^] while releasing the pollen with an estimated peak linear launch acceleration of 2,500*g* and a peak launch velocity of 237 m/s (mean: 170 m/s) [74] (Fig 13C and 13D).

### Osmosis: Water evaporation

#### Elastic Energy Storage in Cell Wall—Fracture Release Mechanism: Drying Coiling Catapult

**Fig 14:**
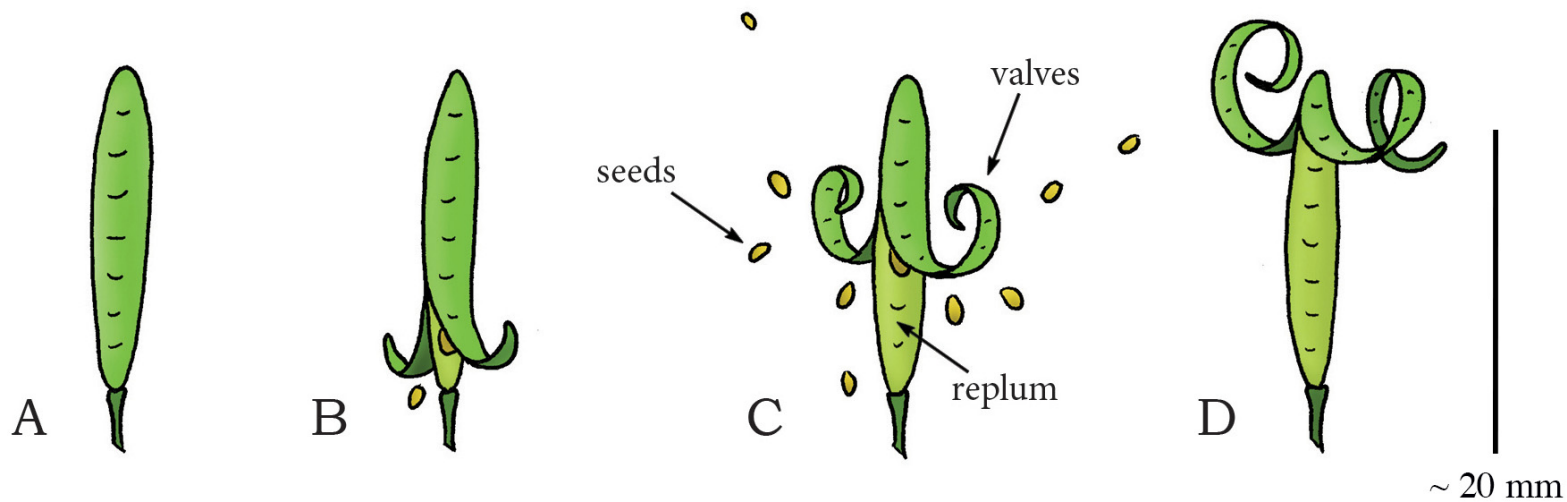
Drying coiling catapult mechanism in *Cardamine parviflora* (Brassicales). (A) Immature untriggered seedpod. (B) Early stage of dehiscence of the seedpod with two valves starting to coil outwards. (C) When a critical pressure is reached, the valves coil rapidly outwards, discharging the seeds. (D) The seedpod after discharge. Drawings based on schematic drawings in [75]. Scale bar 20 mm.

In the mustard family Brassicaceae (phylum: Tracheophyta, class: Magnoliopsida, order: Brassicales) a wide variety of seed dispersal methods are found, including the drying coiling catapult in the genus *Cardamine*, commonly known as bittercress. *Cardamine* is a large genus of over 150 flowering plant species growing in diverse habitats all over the world. In this genus, reproductive units are catapulted by a coiling motion of the plant cells (without fluidic discharge) [75]. The fruit of *C*. *parviflora*, a winter annual that grows up to 35 mm tall, consists of two valves, with the seeds being adhered on the thin internal wall (called the replum) separating the valves (Fig 14A and 14B). During dehiscence, the valves coil outwards in approximately 4.7 ms [75], exposing and launching the seeds (mass of 0.15 mg [75]) with a peak launch velocity of 12 m/s (mean: 6.3 m/s) and a mean launch angle of 52.2° to the horizontal, resulting in a peak launch distance of about 2 m (mean: 0.94 m) (Fig 14C and 14D) [75]. The coiling of the valves in *C*. *parviflora* is presumably driven by a bi-layered cell-structure that stores an energy amount of approximately 89 J/kg, with energy transfer efficiency to the seeds of 21.3% [75] (cf. the bi-layered cell-structure hypothesized for *I*. *capsensis* in [69] and presented above). However, disagreement exists about whether drying or swelling is the driving force in the explosive seed dispersal in *Cardamine*. Based on high-speed video analysis and mechanical energy storage measurements (calculated from the integral of the force-length relationship in the valves as measured with a force transducer), Hayashi *et al*. [75] suggest that the valve curling is driven by the absorption of water. Based on electron microscopic images of the cell structure, Vaughn *et al*. [76], on the other hand, argue that the tension built in *Cardamine* is generated upon drying and explicitly refute the model proposed by Hayashi *et al*. [75]. Because Vaughn *et al*. [76] provide more compelling evidence based on the cell structure found in the valves, we classified this mechanism into the water evaporation category. More research is warranted to precisely determine the working principle of the shooting mechanism in *Cardamine*.

**Fig 15:**
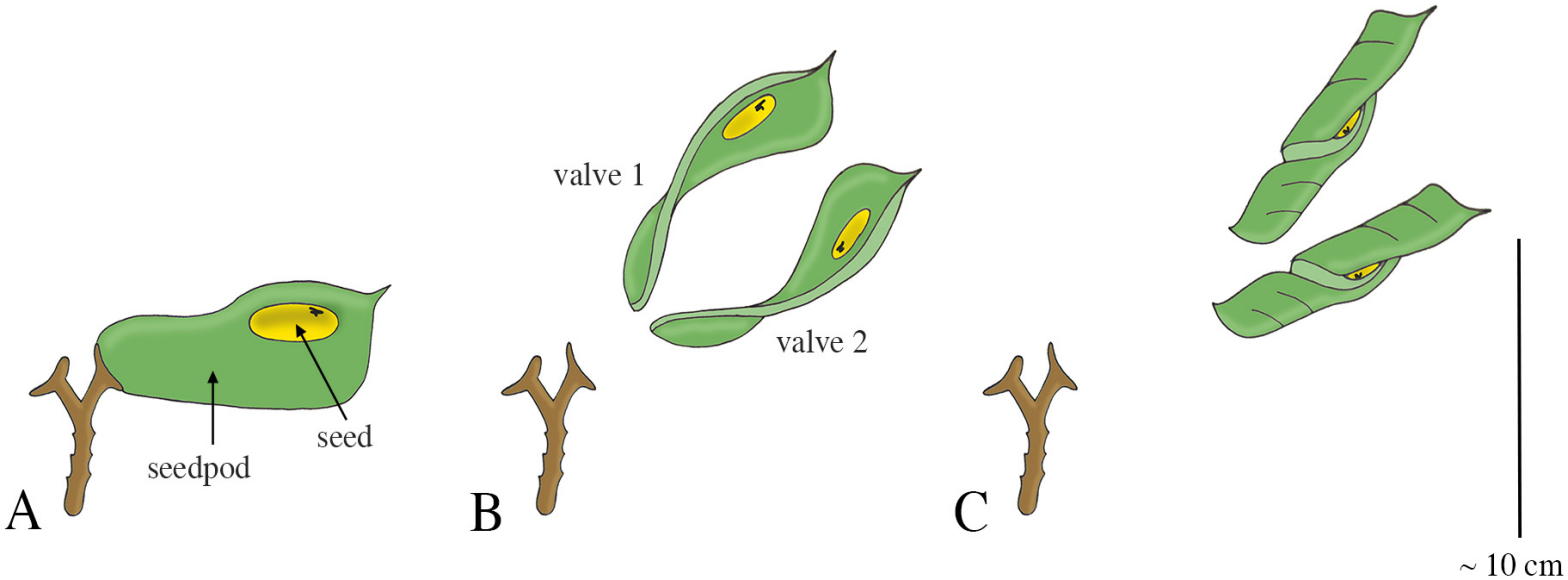
Drying coiling catapult mechanism in *Tetraberlinia moreliana*. (A) A fruiting pedicel with one mature seedpod, consisting of two valves (right) and one exploded seedpod (left). Drying of the valves causes tension in the connection between the valves and stalk of the fruiting pedicel, as the preferred dry shape of the valves is helical. (B) Dehiscence of the valves discharges the seeds. (C) Fully dried valve with seed. Drawings based on schematic drawings in [78]. Scale bar 10 cm.

Similar drying coiling catapult mechanisms as observed in *Cardamine* are found in the Fabaceae (phylum: Tracheophyta, class: Magnoliopsida, order: Fabales), commonly known as the legume, pea, or bean family. Fabaceae is the third largest land plant family of economically important flowering plants, including trees, shrubs, and herbaceous plants. An example of a drying coiling catapult is found in the legume species *Cytisus multiflorus*, commonly known as the white Spanish broom. The fruit of *C*. *multiflorus* is a hairy legume pod (resembling a pea pod up to 3 cm in length), which contains four to six seeds. Desiccation of the pod creates tension in different cell layers at different angles. After a critical tension is reached, explosive dehiscence of the pod discharges the seeds at launch distances of up to 4 m [77]. The legume *Tetraberlinia moreliana* uses a slight variant of the drying coiling catapult mechanism. The mature seedpods (containing on average two seeds; mean seed mass 2.53 g) of this rainforest tree are woody structures resembling the shape of a dragonfly wing (Fig 15A) [78]. The seedpod consists of two valves enveloping the seeds. Drying of the pods causes tension between the valves, as they would deform into a helical shape without constraints (representing a minimum in the elastic energy content). When a critical pressure is reached, the valves break apart and rapidly coil into a helical shape, discharging the seeds with a peak launch velocity of 70 m/s (mean: 37 m/s), and a peak launch distance of 60 m with a mean launch angle of 17.3° to the horizontal (Fig 15B and 15C) [78]. According to Van der Burgt *et al*. [78] the seeds were not significantly influenced by the wind or aerodynamic lift force during the free flight phase of their experiment and are most likely not dispersed by animals. The measured dispersal distance is thus a direct derivative of the ballistic dispersal process (and the encountered viscous drag). However, based on the shape of the valves, we hypothesize that aerodynamics force may play a significant role and wind dispersal is a real possibility. Finally, the legume *Bauhinia purpurea* also ballistically disperses its seeds [79], but its mechanism was not found in literature.

Hildebrand [80] was the first to describe the structure of the fruits of Acanthaceae (phylum: Tracheophyta, class: Magnoliopsida, order: Lamiales) that enables them to ballistically disperse their seeds. The seed capsules of the Acanthaceae subfamily Acanthoideae can either be discharged using water absorption or water evaporation, but in both cases the mechanism for discharge is similar [81]. Witztum *et al*. [81] describe the working mechanism of *Ruellia simplex* (synonym *Ruellia brittoniana*), also known as Britton’s wild petunia. In this species, the seed capsule consists of two interconnected slender valves enveloping 16–20 seeds (mean seed mass 1.78 mg). A valve consists of three main layers; an inner “resistant” cell-layer (when it dries it only shrinks very minimally), a middle “inert” layer, and an outer “active” cell-layer that shrinks considerably by water evaporation [81] (similar to a bimetal [81]). As the deformation of the adjacent valves is obstructed by their connection, the water evaporation from the valves tensions the active layer. Drying of the capsule thus results in the storage of elastic energy in the valves. Dehiscence of the connection holding the two valves together releases the elastic potential energy stored in the valves and transfers it into kinetic energy of both the valves and seeds. In *Ruellia simplex*, dehiscence is due to moisture absorption of the capsule beak (after the capsule has dried), which weakens the pectic “glue” that holds the valves together, whereas in other species dehiscence is due to the high stress in the bonding layer. The seeds of *R*. *simplex* are thrown for distances of up to 3 m with a launch angle of 40° [81]. It is suggested by Witztum *et al*. [81] that the mechanical design is optimized to increase the launch distance of the seeds by an optimal cross-sectional area division of the tissue types in the active and resistance layers, the presence of the “inert” layer, and the use of “jaculators” that optimize the launch angle of the seeds. Another Acanthaceae species that actively disperses it seeds is *Acanthus mollis* [81].

**Fig 16:**
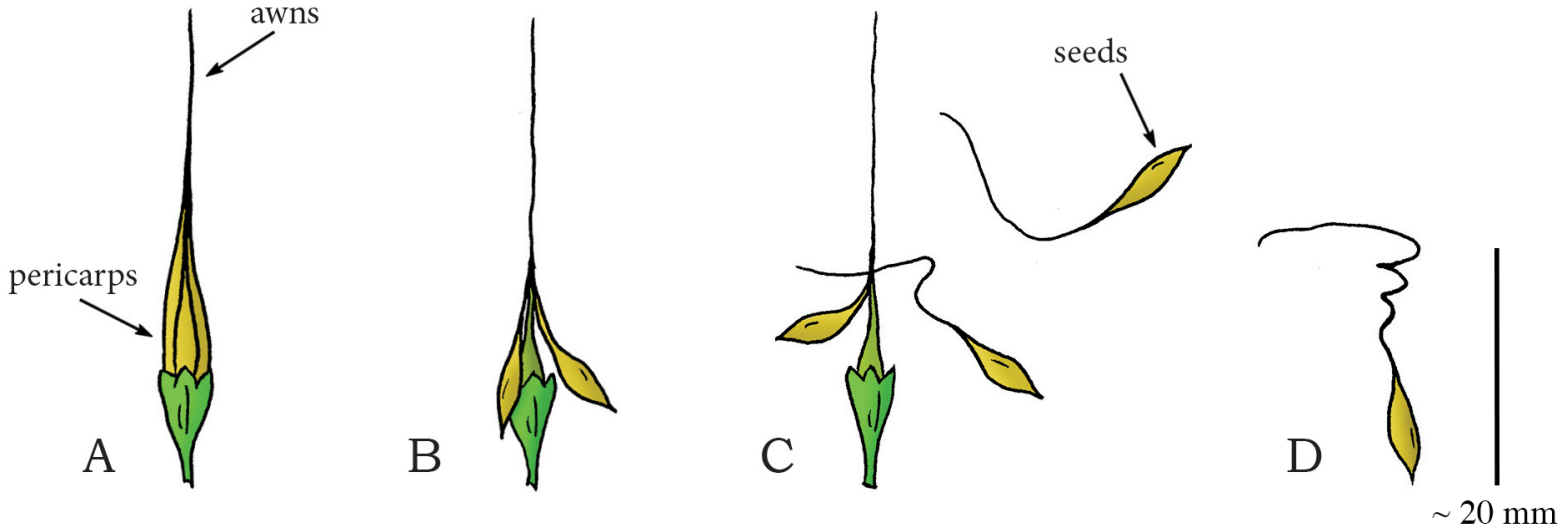
Drying coiling catapult mechanism in the geranium *Erodium cicutarium*. (A) Fruit consisting of five interconnected pericarps with long awns. (B) Dehydration of the awns creates tension in the awns, as the preferred dry shape of the awns is helical, resulting in dehiscence. (C) When a critical stress is reached, complete dehiscence of the connection between the awns discharges the seeds. (D) A discharged dry seed with awn. Drawings based on a photograph in [82]. Scale bar 20 mm.

In Geraniaceae (phylum: Tracheophyta, class: Magnoliopsida, order: Geraniales), a family of flowering plants including the genus *Geranium*, multiple ballistic shooting mechanisms for seed dispersal are observed [62]. Stamp *et al*. [62] investigated three species of wild geranium (*G*. *carolinianum*, *G*. *maeulatum*, and *G*. *molle*) in terms of seed dispersal distance and seed morphology. Mean seed dispersal distances of 3.29 m, 3.02 m, and 1.79 m have been observed in *G*. *carolinianum*, *G*. *maeulatum*, and *G*. *molle*, respectively. Evangelista *et al*. [82] describes the working mechanism of another geranium species: *Erodium cicutarium*, commonly known as common stork’s-bill and pinweed. The fruit of this species consists of five interconnected valves (pericarps), containing seeds (mean mass of 5 mg) with so-called awns (i.e. hair- or bristle-like appendages of the seed) (Fig 16A) [82]. The preferred dried shape of the cellular structure of the awn is helical, inducing tension in the joined structure of the valves upon drying of the awns. When a critical tension is reached, the joined structure fractures, which releases and subsequently discharges the awns at a mean launch angle of 40° to the horizontal with a peak launch velocity of 10 m/s (mean: 4 m/s), resulting in a peak launch distance of 0.75 m (mean: 0.51 m) (Fig 16B–16D) [82].

#### Elastic Energy Storage in Cell Wall—Fracture Release Mechanism: Drying Squeeze Catapult

**Fig 17:**
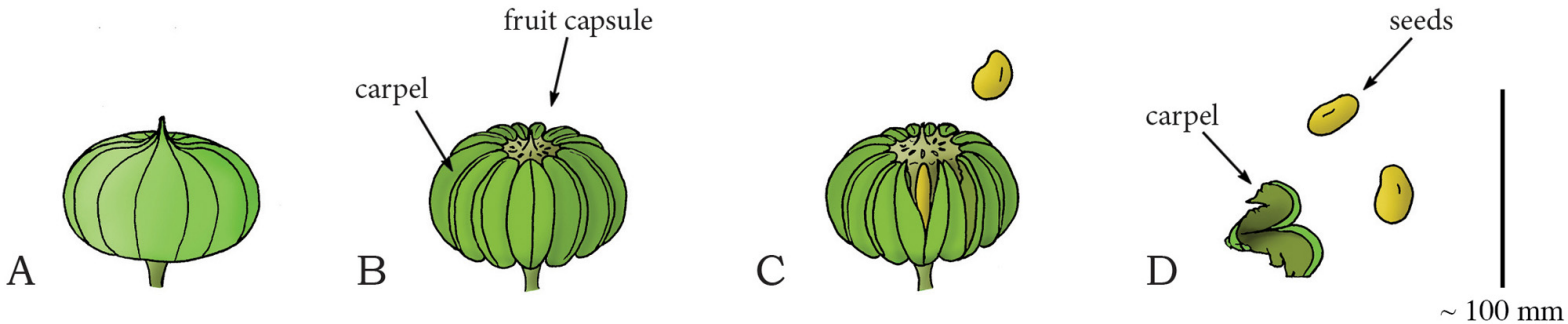
Drying fracture catapult mechanism in the *Hura crepitans*. (A) The immature fruit of *H*. *crepitans*. (B) The full-grown fruit, consisting of several carpels embracing the seeds. (C) When a critical pressure is reached, the carpels split open from the central axis, discharging the seeds. (D) A separated carpel and launched seeds. Drawings based on schematic drawings in [79]. Scale bar 100 mm.

Within the spurge family of Euphorbiaceae (phylum: Tracheophyta, class: Magnoliopsida, order: Malpighiales), several species, including *Hura crepitans* and *Mercurialis annua*, are known for their active seed dispersal mechanism. *H*. *crepitans*, native to tropical regions of North and South America, including the Amazon, is an evergreen spurge tree, which contains long dark spines and a smooth bark. *H*. *crepitans* is also known as monkeys’ dinner bell and monkeys’ pistol because of the loud sound made by the fruit capsule during dehiscence, signaling the monkeys that it is time to eat. The fruit of this species is a pumpkin-shaped capsule, consisting of several compartments (carpels) arranged around a central axis (Fig 17A and 17B) [79]. A slightly different geometry of the fruit capsule is observed in the spurge *M*. *annua*, a small annual herb native to Europe, North Africa, and the Middle East. The fruit of *M*. *annua* resembles two interconnected spheres, each containing one seed (mass 0.6–3.6 mg), with a suture line running across each of the spheres [83]. *H*. *crepitans* and *M*. *annua* both catapult their reproductive units by a “squeeze” force generated by water evaporation in the plant cells. Dehydration-induced tension in the different layers of cells of the fruit wall causes the carpels to separate from the central axis and split into two halves that eject the seeds (mean mass 1,020 mg in *H*. *crepitans*). In *H*. *crepitans*, a peak launch velocity of 70 m/s (mean: 43 m/s), a peak launch distance of 45 m (mean: 30 m), and mean launch angle of 34.2° to the horizontal was found [79,84] (Fig 17C and 17D). For *M*. *annua*, a peak launch distance of 1.3 m (mean: 0.41 m) is reported [83]. The energy storage mechanism and the specific cell structure responsible for the shooting action of both species have not yet been unraveled fully.

Multiple species of the Rutaceae family (phylum: Tracheophyta, class: Magnoliopsida, order: Sapindales), for example *Metrodorea nigra* [85], the genus *Illicium* (phylum: Tracheophyta, class: Magnoliopsida, order: Austrobaileyales, family: Schisandraceae), for example *Illicium floridanum* (mean seed mass 45 mg) [86], and *Oxalis acetosella* (phylum: Tracheophyta, class: Magnoliopsida, order: Oxalidales, family: Oxalidaceae (woodsorrels)) (mean seed mass 0.9 mg) [87] also use a drying squeeze catapult for discharging their seeds. Similar to *H*. *crepitans*, the fruits of these families and genera generally consist of multiple interconnected carpels that are radially arranged from a central stalk. During development, the fruits show small splits along the line of separation between the carpels or on the carpel surface. Dehydration of the carpels creates tension along the weakest regions (i.e. the connections between the carpels or along a so-called suture line running along the circumference of the carpels themselves), which eventually causes dehiscence, splitting the carpels into two halves or the carpels from the central stalk. The seeds are subsequently discharged with a launch angle of 40–60° to the horizontal, resulting in a peak launch distance of 5.8 m (mean: 2.5 m) in *I*. *floridanum* [86]. Furthermore, a peak launch distance of 2 m was found in *O*. *acetosella* [87].

Ballisitic seed dispersal occurs also in *Viola* (phylum: Tracheophyta, class: Magnoliopsida, order: Malpighiales, family: Violaceae), a genus of flowering plants, which share a remarkably similar floral structure [62,88]. Two example species with active seed dispersal are *V*. *eriocarpa* and *V*. *striata* [62]. Their shooting mechanism was suggested to be similar to a marble being squeezed by the fingers [88]. A peak launch distance of 5.4 m was found in *V*. *eriocarpa* [88].

#### Elastic Energy Storage in Air and Cell Wall—Fracture Release Mechanism: Air Pressure Catapult

**Fig 18:**
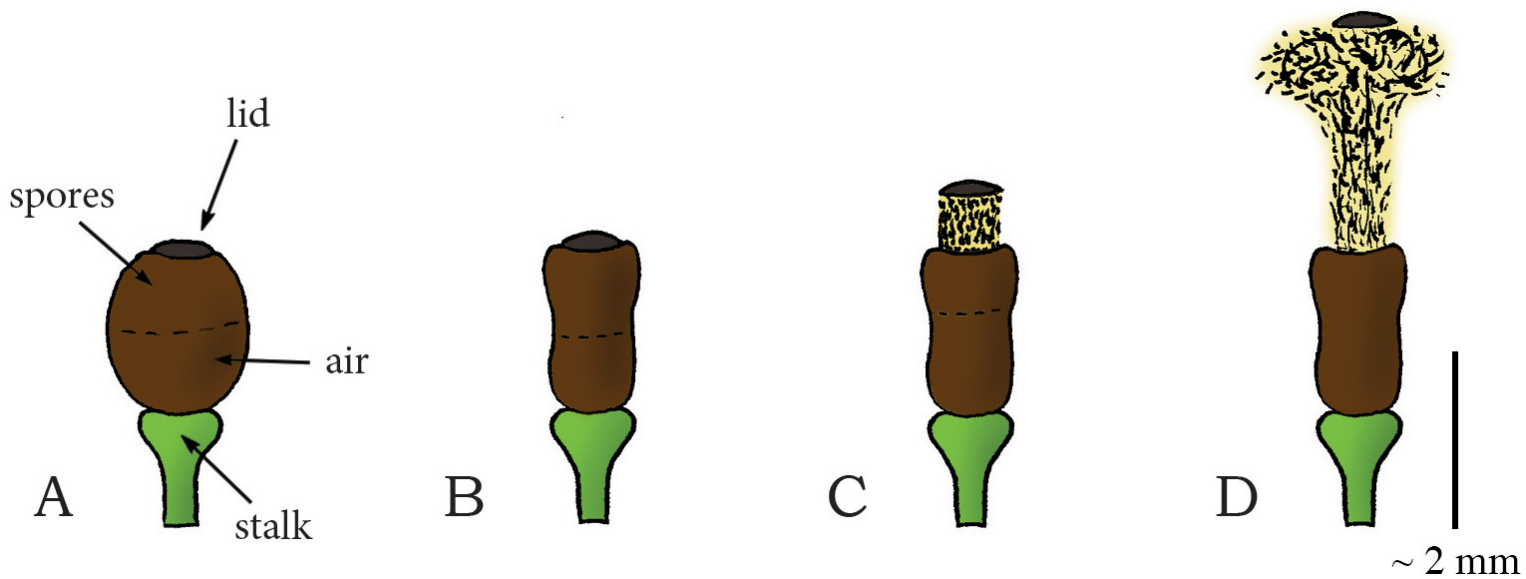
Air pressure catapult mechanism in the genus *Sphagnum*. (A) The mature spherical spore capsule of *Sphagnum* filled with spores and air (equal portions). (B) Deformation of the capsule into cylindrical shape due to drying, which raises the air pressure in the capsule. (C–D) When a critical pressure is reached, sudden fraction of the capsule lid explosively discharges the spores from the capsule. Drawings based on schematic drawings in [1]. Scale bar 2 mm.

In *Sphagnum* (phylum: Bryophyta, class: Sphagnopsida, order: Sphagnales, family: Sphagnaceae), a genus of approximately 120 species of mosses, generally known as peat moss, reproductive units are catapulted by an air jet. The spores of *Sphagnum* are developed within spherical capsules grown on short stalks [89]. The spherical capsule comprises two parts: an upper spore-filled chamber and a bottom air-filled chamber (Fig 18A). The capsule wall consists of four to five layers of cells, delimited by a circular line along the operculum rim, which circumscribes the lid of the capsule [89] (Fig 18A). The mature capsule is much darker than the stalk; it absorbs light relatively well, which promotes heating and drying of the capsule on sunny days. The mature drying capsule contracts radially, transforming the capsule shape from spherical to cylindrical [33,90,91] (Fig 18B). Both the circumference and volume of the capsule reduce, raising the air pressure in the bottom air-filled chamber until a critical pressure (estimated between 0.2 and 0.5 MPa [1,33,90]) is reached. Fast release of the lid triggers explosive spore discharge by the internal air pressure, at a peak acceleration of 36,697*g* [1] and a peak launch velocity of 30 m/s (mean: 18 m/s) [90] that propels the spores (20,000 to 240,000 spores per capsule) with an estimated launch angle 80–90° (as measured from a figure in [89]) as high as 0.20 m above the moss (Fig 18C and 18D) [89,90,92]. As we discussed in the section on ascomycetes, the collective discharge of many spores is required to minimize the effect of viscous drag, enabling spores to travel larger distances [1]. In this shooting mechanism, the energy is stored primarily in the compressed air, as well as in the cell wall. The explosive mechanism is no longer observed when the air chamber in the spore capsule is punctured, as this reduces the pressure in the chamber to the ambient value. Nevertheless, the spores can be released slowly from punctured capsules because the lid (partly) separates from the capsule at large radial contractions of the capsule [91]. We note an important difference here compared to shooting mechanisms that rely on the compressive liquid-filled containers discussed above: since a liquid is nearly incompressible (bulk modulus is about 2·10^**9**^ Pascal [Pa = N/m^**2**^] for water; for air 10^**5**^ Pa), only a small amount of energy can be stored in the liquid, whereas in *Spagnum* the main energy storage used for shooting is in the compressed air.

#### Elastic Energy Storage in Cell Wall—Cavitation Release Mechanism: Cavitation Coiling Catapult

**Fig 19:**
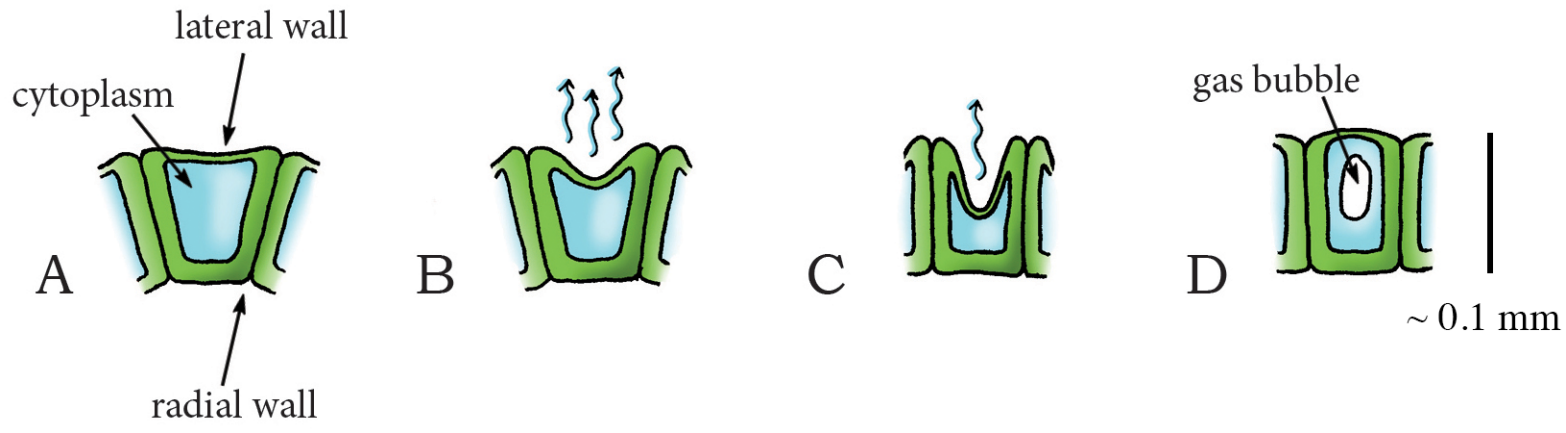
Cavitation in plant cells. A regular plant cell consisting of a cell wall and cytoplasm. (B) Evaporation of water from the cell causes the radial walls to come closer together and the lateral wall to cave inwards. (C) The lateral wall is caved inwards completely. (D) When a critical pressure is reached, the cytoplasm fractures and a gas (cavitation) bubble appears, causing the walls to rapidly snap back to their original form. Drawings based on figures in [95]. Scale bar 0.1 mm (100 μm).

**Fig 20:**
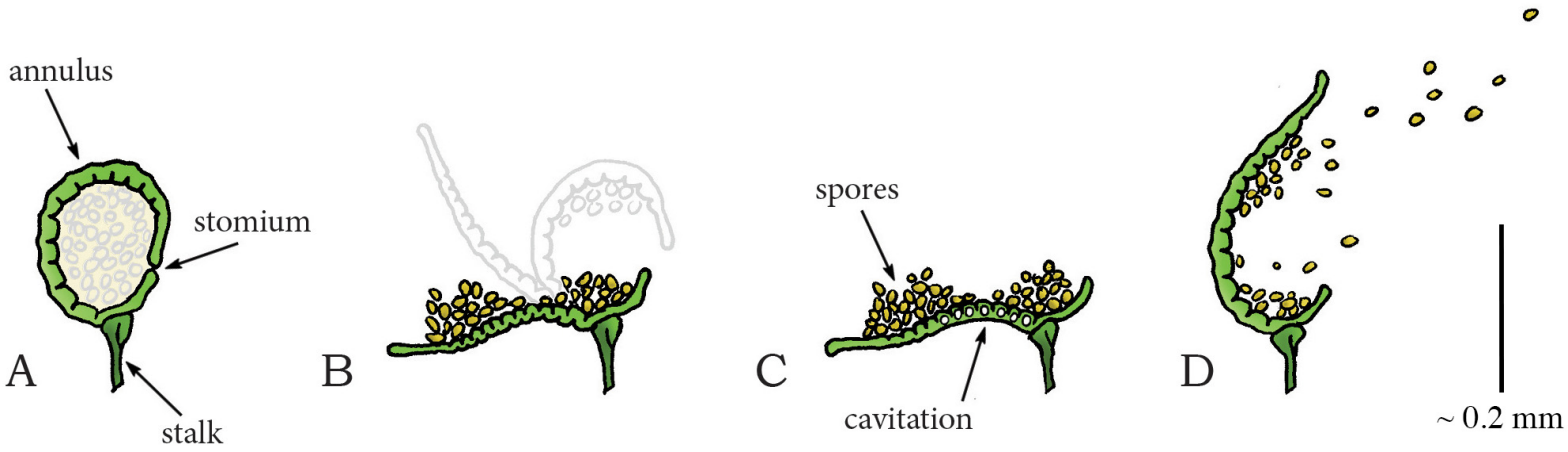
Cavitation catapult mechanism in the family Polypodiaceae or common ferns. (A) The mature sporangium in common ferns consisting of a stalk and an annulus enclosing the spores. (B) Dehydration of the annulus cells causes the radial cell walls to come closer together and the lateral walls to collapse internally, straightening the annulus. (C) When a critical pressure (between -9 and -20 MPa relative to ambient) is reached, cavitation occurs in the cells of the annulus. (D) Discharge of the spores by quick release of the elastic energy stored in the cell walls as the annulus snaps back to its original shape. Drawings based on high-speed images in [94]. Scale bar 0.2 mm (200 μm).

Cavitation-based spore discharge, similar to that of fungi imperfecti, is also observed in the family Polypodiaceae (phylum: Tracheophyta (Pteridophyta), class: Polypodiopsida/Pteridopsida, order: Polypodiales), also known as common or polypod ferns (e.g. the species *Adiantum peruvianum* [93] and *Polypodium aureum* [94]) [8,37,94,95]. Most species in the family Polypdiaceae are epiphytes (i.e. plants that grow harmlessly on another plant and derive water and nutrients directly from the air, rain, and decaying material in their surroundings). The spore-bearing structure of common ferns consists of a stalk and an annulus of twelve or thirteen cells forming a circular crest that encloses the spores (Figs 19 and 20A). Evaporation of water from the cells’ cytoplasm brings the radial walls closer together and makes the lateral walls collapse internally (Figs 19A–19C and 20A–20C), causing the annulus to open at the stomium (Fig 20A) and expose the spores (Fig 20B) [93]. When the water tension reaches a critical negative value of -9 to -20 MPa relative to ambient [8,93], the cytoplasm fractures and cavitation occurs within adjacent cells (Figs 19D and 20C), leading to a quick release of the stored elastic energy of the cell walls as the annulus snaps back to its original shape (~0.01 ms [94]). This rapid motion catapults the spores with a peak launch acceleration of approximately 100,000*g* and a peak launch velocity of 10 m/s in *Polypodium aureum* (Fig 20D) [94,95]. From data in [93], we estimated a peak spore launch distance of 0.057 m in *A*. *peruvianum*.

A similar pollen dispersal mechanism as those in common ferns is observed in representatives of the genus *Selaginella* (phylum: Tracheophyta (Lycopodiophyta), class: Lycopodiopsida, order: Selaginellales, family: Selaginellaceae), such as in *Selaginella martensii*, a spikemoss that is native to Mexico and Central America and in which both microspores and megaspores are actively dispersed [96]. *S*. *martensii* has ascending stems with spore-bearing structures, or sporangia, born on the top surface of a modified leaf or sporophyll and clustered together into cones, also known as strobili, approximately between a few centimeters and 30 cm above the soil surface [96]. Active dispersal of the microspores is due to cavitation-induced drying of the valves of the microsporangium. Due to drying, the two valves separate along the dehiscence line and bend away from each other up to an angle of 150° [96]. When the water tension reaches a critical negative pressure, cavitation occurs within the valves, leading to a quick release of the stored elastic energy as the valves snap back to their original shape and catapult the microspores with a peak launch velocity of 0.6 m/s, resulting in a peak launch distance of 6 cm [96]. Presumably, the compressed air between the valves results into a jet that helps to disperse the microspores. Additionally, Schneller *et al*. [96] noticed that thousands of microspores are discharged simultaneously, allowing for a greater dispersal distance and the crossing of the boundary layer by negating the range constraints imposed by viscous drag just as in ascomycetes and *Sphagnum*. The dispersal mechanism of megaspores of *S*. *martensii* is slightly different and consists of two main steps. First, the lower boat-like part of the spore-bearing structure (comprising two valves) constricts upon drying, pressing the two upper spores together until they are discharged, similar to the drying squeeze catapult (see above). Next, the bases of the lower boat-like part of the spore-bearing structure clash together and eject the second spore pair (mean mass 1.4·10^−3^ mg). The spores are ejected with a peak launch velocity of 4.5 m/s, resulting in a peak launch distance of 0.65 m (mean: 0.21 m) [96]. The estimated impulse of one megaspore is 6.3 picoNewton second [pN·s] [96].

## Animals

**Fig 21:**
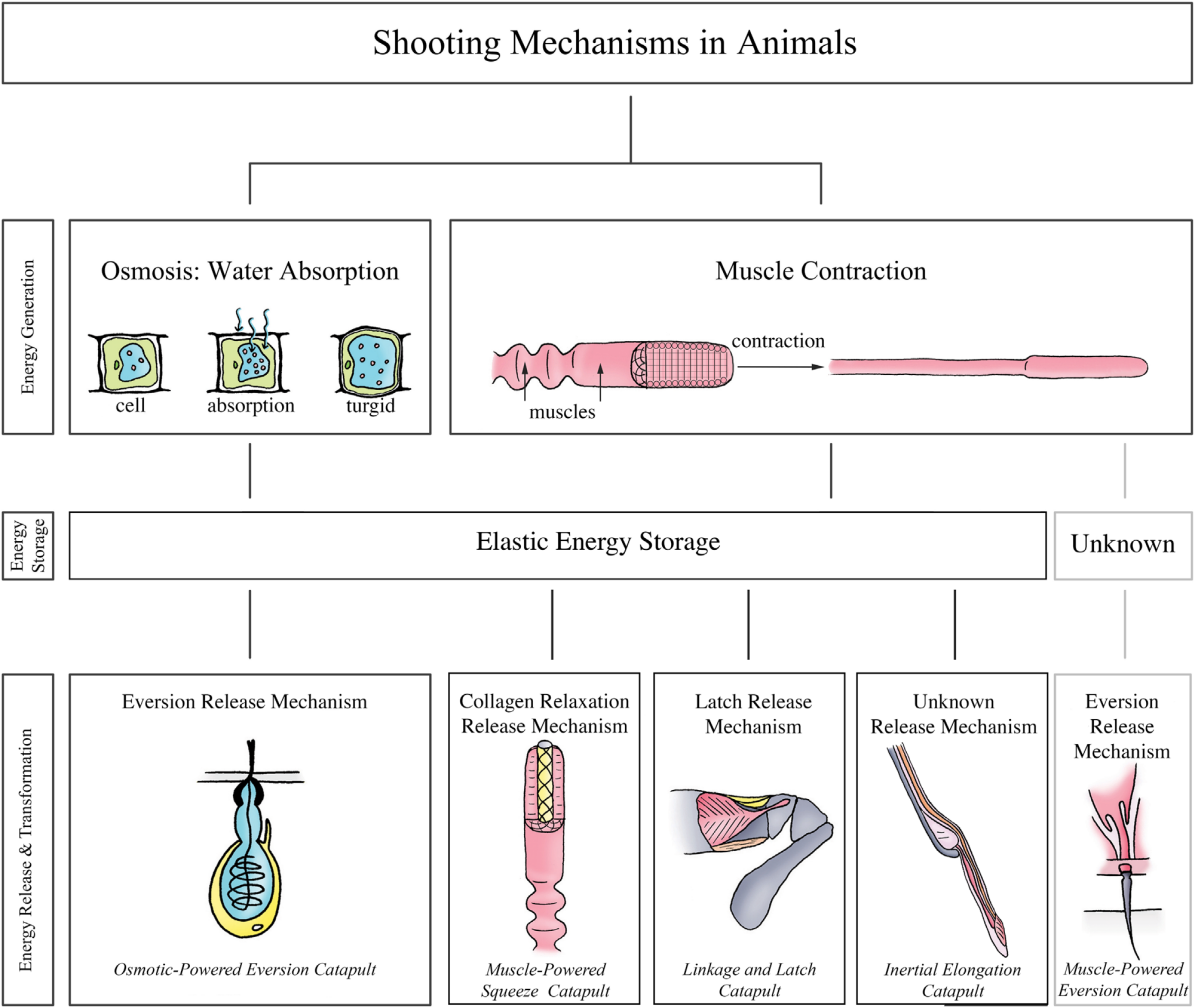
The structural categorization of the identified shooting mechanisms in animals, allocated based on the energy management criteria discussed above. **Osmotic-powered eversion catapult**: observed in the phylum Cnidaria. **Muscle-powered squeeze catapult**: observed in the family Chamaeleonidae, schematic illustration of *Chameleo calyptratus*. **Linkage and latch catapult**: observed in the order Stomatopoda, schematic illustration of *Odontodactylus scyllarus*. **Inertial elongation catapult**: observed in the families Bufonidae, Microhylidae, Dendrobatidae, Megophryidae, Leptodactylidae, and Ranidae within the order Anura, schematic illustration of *Bufo marinus*. **Muscle-powered eversion catapult**: observed in the families Ariophantidae, Bradybaenidae, Dyakiidae, Helicidae, Helminthoglyptidae, Hygromiidae, Parmacellidae, Urocyclidae, and Vitrinidae, within the clade Stylommatophora.

Fig 21 illustrates the shooting mechanisms identified in animals, allocated based on the energy management criteria discussed above. In contrast to the plants and fungi, where shooting mechanisms are primarily used for increasing reproductive success, in the animal kingdom, shooting mechanisms have also evolved for prey capture, locomotion, and anti-predator defense. An overview of all retrieved launch parameters and the associated measurement methods of the identified shooting mechanisms in animals is provided in Table 3.

**Table 3:** Summary of launch parameters of the identified shooting mechanisms in Animals. Abbreviations: BL = body length, ML = mandible length, SL = skull length, SD = strike distance, RL = resting length, BM = body mass, PV = (time to) peak velocity, PA = (time to) peak acceleration. For the projectile mass, launch velocity, launch acceleration, launch distance, and power output, the measurement technique is indicated as: Standard = measured using a HSV camera. **Bold** = calculated by referred authors using measured launch parameters. *Italics* = manual measurement of the parameter (e.g. from photograph stills, without the use of a high-speed video camera). Standard* = estimated by us from data/figure in indicated reference(s); for the power output per unit mass the launch acceleration [m/s^2^] is multiplied with the launch velocity [m/s] and for the work per unit mass the power output [W/kg] is integrated over the launch duration [s]. On some occasions the time to peak acceleration or time to peak velocity is used to calculate the work per unit mass delivered by the shooting mechanism, this is indicated with PA and PV, respectively. Bold* = calculated by us using the mean parameter ± 3 standard deviations. The launch parameters are indicated as mean (± standard deviation), peak (indicated with “peak” behind the value), or a range (minimum value–maximum value). Per launch parameter, the peak value identified in this review is indicated by a double-lined box.

Animals	Projectile mass [g]	Launch acceleration [*g*]	Launch velocity [m/s]	Launch distance [m]	Power output [W/kg]	Launch duration [ms]	Work [J/kg]
Osmosis: Water Absorption							
**Elastic Energy Storage—Eversion Release Mechanism**							
Osmotic-powered Eversion Catapult							
Cnidaria (cnidarians) [2,29,102,103,105]	*1*·*10*^*−9*^*–2*.*3*·*10*^*−9*^	5.41·10^6^ peak	18.6 (37.1 peak)	0.0006 peak	1.97·10^9^ peak*	0.0021*	4,137*
Muscle Contraction							
**Elastic Energy Storage—Geometrical Release Mechanism**							
Muscle-Powered Squeeze Catapult							
Iguania (iguanian lizards)							
Chamaeleonidae (chameleons) [117]				200% BL peak			
*Bradypodion melanocephalum* [3]	-	93.8–112.1	3.9–4.3	0.079–0.083	**4,920–5,840**	19.3–23.3	95–135.5*
*Bradypodion occidentale* [3]	-	57.2–57.6	3.9–4.2	0.094–0.12	**2,880–3,160**	25.3–28	72.9–88.5*
*Bradypodion pumilum* [3]	-	60–82.9	3.7–3.9	0.084–0.12	**3,020–4,140**	26.7–34.7	80.6–143.7*
*Bradypodion sp*. *“emerald”* [3]	-	53.8–96.2	3.7–4.6	0.11–0.15	**2,600–5,720**	29–34	75.4–194.5*
*Bradypodion thamnobates* [3]	-	50–92.5	2.9–4.3	0.10–0.14	**1,802–4,680**	20–38.7	36–181.1*
*Brookesia superciliaris* [3]	-	137.6–142.7	4.4–5	0.07–0.09	**7,920–9,080**	17.7–19.7	140.2–178.9*
*Calumma p*. *parsonii* [3]	-	42.5 peak	4.9 peak	0.2 peak	**2,880 peak**	39 peak	112.3 peak*
*Chameleo calyptratus* [3,8,26,30,115]	-	41 (52.4 peak)	3.9 (5.0 peak)	0.13 (0.07–0.32)	**1,092 (3,480 peak)**	13–48	14.2–167*
*Chameleo jacksonii* [112]	-	17 peak	3.7* (6.5 peak*)	0.097 ± 0.002	608.7 peak*	27.8 ± 2	21.7 peak*
*Chameleo melleri* [30,34]	*4*	38.1 ± 2.5 (40.8 peak)	6 peak	-	**1,584 ± 176 (3,000 peak)**	~20	31 (60 peak)*
*Chameleo pardalis* [8,30]	-	34.7 ± 3.4	5 peak	-	**1,170 ± 176 (3,000 peak)**	~20	23.4 (60 peak)*
*Furcifer lateralis* [3]	-	74 peak	4.1 peak	0.11 peak	**4,080 peak**	32.3 peak	131.8 peak*
*Furcifer oustaleti* [3]	-	29.2–46.3	3.6–4.8	0.18–0.27	**1,410–2,980**	47.3–54.6	66.7–162.7*
*Kingyongia fischeri* [3]	-	59.1–67.4	4.5–4.8	0.177–0.179	**3,820–4,420**	37–41.3	141.3–182.6*
*Kingyongia tenuis* [3]	-	116.2 peak	4.9 peak	0.1 peak	**7,820 peak**	12.3 peak	96.2 peak*
*Rhampholeon acuminatus* [3]	-	119.3–132.5	4.8–5.2	0.082–0.097	**7,720–8,840**	22.7–23.3	175.2–206*
*Rhampholeon spinosus* [3]	-	180.4–264	5.1–5.3	0.11–0.12	**12,100–14,040**	18.3–22.7	221.4–318.7*
*Rieppeleon brevicaudatus* [3]	-	111.1–165.1	3.6–5.4	0.037–0.1	**5,120–11,620**	9.7–18.7	49.7–217.3*
*Trioceros cristatus* [3]	-	77.8 peak	4.7 peak	0.17 peak	**5,220 peak**	36.7 peak	191.6 peak*
*Trioceros hoehnelii* [3]	-	76.6–77.2	4–4.3	0.11–0.2	**3,480–4,500**	31–48.3	107.9–217.4*
*Trioceros jacksonii xantholophus* [3]	-	65.3 peak	4.4 peak	0.16 peak	**4,140 peak**	34 peak	140.8 peak*
*Trioceros johnstoni* [3]	-	62 peak	4.6 peak	0.19 peak	**4,000 peak**	47.7 peak	190.8 peak*
*Trioceros montium* [3]	-	68.3–71.5	4.2–4.5	0.16–0.18	**3,920–4,560**	33.3–39.3	130.5–179.2*
Plethodontidae (lungless salamanders) [117]	*~ 1*			80% BL peak			
*Bolitoglossa dofleini* [31]	*0*.*79*	177 ± 11.1 (458 peak)	3.8 ± 0.1 (7 peak)	0.02 (0.03 peak)	**4,109 ± 424 (18,129 peak)**	7 ± 0.4 (4 ± 0.3 PA)	28.8*(16.5–44.1*)(6.4 ± 0.5 PA)
*Eurycea guttolineata* [31,128]	*1*.*29*	80.7 ± 6.5 (105.6 peak)	2.3 ± 0.2 (3.2 peak)	0.009 (0.02 peak)	**1,778 ± 229 (2,467 peak)**	7 ± 0.4 (4 ± 0.3 PA)	12.4*(6.3–20.2*)(4.1 ± 0.3 PA)
*Eurycea wilderae* [31]	-	140.4 ± 10.6 (203.1 peak)	2.5 ± 0.1 (3.2 peak)	0.009 (0.02 peak)	**2,818 ± 322 (5,921 peak)**	7 ± 0.4 (4 ± 0.3 PA)	19.7*(10.7–48.6*)*)(4.1 ± 0.3 PA)
*Hydromantes genei* [31]	-	102.9 ± 5.9 (119.7 peak)	4.6± 0.1 (4.9 peak)	0.03 (0.04 peak)	**1,860 ± 330 (4,305 peak)**	13 ± 1 (6 ± 1 PA)	24.2*(8.7–45.6*)(6.5 ± 0.9 PA)
*Hydromantes imperialis* [31]	*1*.*04*	82.6 ± 11 (93.6 peak)	4.0 ± 0.0 (4.2 peak)	0.03 (0.04 peak)	**2,495 ± 428 (2,923 peak)**	13 ± 1 (6 ± 1 PA)	32.4*(12.1–46.8*)(6.5 ± 0.9 PA)
*Hydromantes platycephalus* [31]	-	42.1 ± 6.6 (100.3 peak)	2.7 ± 0.2 (3.7 peak)	0.03 (0.04 peak)	**908 ± 186 (2,443 peak)**	13 ± 1 (6 ± 1 PA)	11.8*(3.5–39.1*)(6.5 ± 0.9 PA)
*Hydromantes supramontis* [31,127]	-	-	-	0.6 (80% BL)	-	-	-
**Elastic Energy Storage—Latch Release Mechanism**							
Linkage and Latch Catapult							
Stomatopoda (mantis shrimps)	-	925–10,601*	5.7–23*	-	5.19·10^4^–4.7·10^5^*	-	-
*Alachosquilla vicina* [131]	-	925 ± 331^a^	5.7 ± 0.91^a^	-	5.19·10^4^ *(1.61·**10**^**5**^ peak*)	3.26± 0.41 (1.1 ± 0.02 PV)	169.2*(722.9 peak*)(57.09*(186.8 peak*) PV)
*Odontodactylus scyllarus* [25,131]	-	6,626–10,600	14–23^a^	0.08 peak SD	**4.7**·**10**^**5**^	2.7 PA	1,269 peak* PA
**Elastic Energy Storage—Unknown Release Mechanism**							
Inertial Elongation Catapult							
Anura (toads and frogs)							
Bufonidae [16]	-						
*Bufo alvarius* [142]	*10*.*3 ± 0*.*89*	-	-	200% RL	-	-	-
Mouth opening value:	-	20.2–80.4	0.7–1.0	-	**2,700–9,600**	2	5.4–19.2*^b^
*Bufo marinus* [16,136,141]	*0*.*75 ± 0*.*05*	25.5–31.6	2.9 peak	0.039 ± 0.002(0.023–0.059)	886.6 peak*	31 ± 1 (25–42)	37.2*
Mouth opening value:	-	-	-	-	-	-	-
Hylidae [16,137]							
*Pachymedusa dacnicolor* [137]	-	-	1.7–2.9*	0.012 ± 0.001	-	7 ± 0.6	-
Mouth opening value:	-	-	-	-	-	50 ± 4.4	-
Microhylidae [138]							
*Dyscophus guineti* [16,138,141]	*0*.*81 ± 0*.*1*	4.9–14.7	1.2 ± 0.07 (2.2 peak)	0.0011 ± 1 10^−4^	168.6–316.8*	16.6 ± 0.9 (6.4±1.2 PV)	2.3–6.1 (0.5–3.2 PV)*
Mouth opening value:	-	-	1.3 ± 0.05	-	-	1.7 ± 0.9 PV	-
Dendrobatidae							
Megophryidae							
Leptodactylidae							
Ranidae							
*Rana pipiens* [139]	*0*.*9 ± 0*.*05*	6.6–45.8	0.6–3.2	0.009–0.038	**25–885**	17–92	0.4–81.4*
Mouth opening value:	-	3.2–10.1	0.1–0.8	-	**115–1,783**	9–24	**0.6–6.3**^c^
**Unknown approach—Eversion Release Mechanism**							
Muscle-Powered Eversion Catapult							
Stylommatophora (snails and slugs) [144–147]							
Ariophantidae	-	-	-	-	-	-	-
Bradybaenidae	-	-	-	-	-	-	-
Dyakiidae	-	-	-	-	-	-	-
Helicidae	-	-	-	-	-	-	-
Helminthoglyptidae	-	-	-	-	-	-	-
Hygromiidae	-	-	-	-	-	-	-
Parmacellidae	-	-	-	-	-	-	-
Urocyclidae	-	-	-	-	-	-	-
Vitrinidae	-	-	-	-	-	-	-

^a^ The angular accelerations [rad/s^2^] and angular speed [rad/s] of the mantis shrimp appendage strike are measured in *Alachosquilla vicina* and *Odontodactylus scyllarus* as:

*A*. *vicina* [131]: 2.58·10^6^ ± 1.04·10^6^ rad/s^2^ and 1.61·10^3^ ± 0.34·10^6^ rad/s

*O*. *scyllarus* [25,131]: unknown and 670–990 rad/s peak

^b^ Total work 4.3 mJ [142]

^c^ Our own calculations show different numbers: 1–42.8 J/kg. Maximum kinetic energy of ballistic opening 0.029–0.28 mJ [139].

### Osmosis: Water Absorption

#### Elastic Energy Storage in Cell Wall—Eversion Release Mechanism: Osmotic-Powered Eversion Catapult

**Fig 22:**
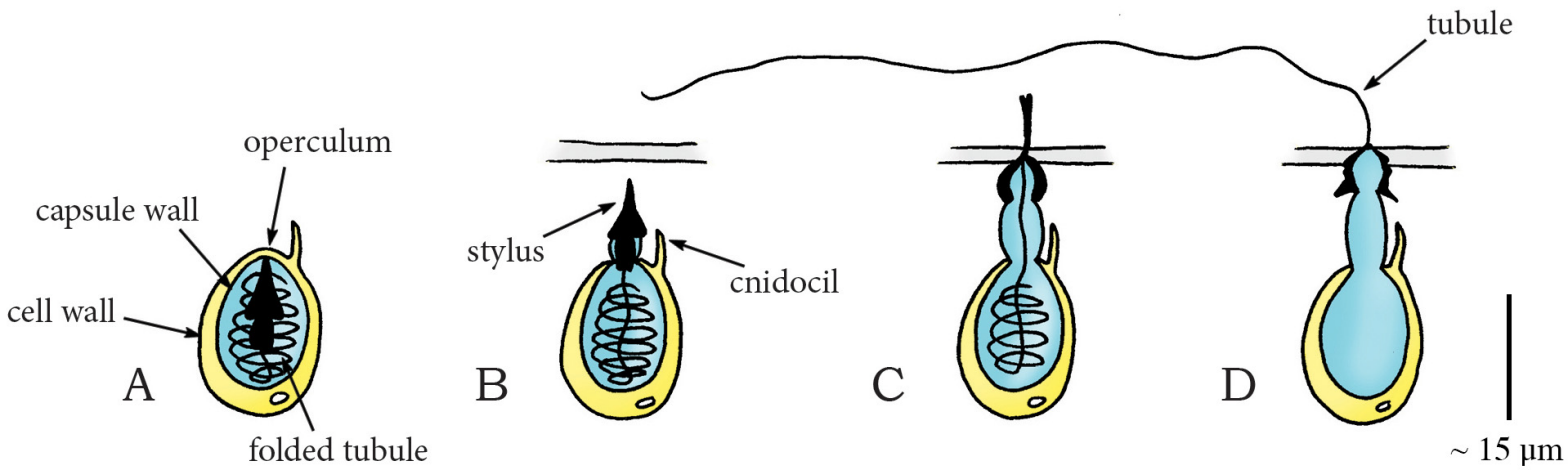
Osmotic-powered eversion catapult mechanism in the phylum Cnidaria. (A) The cnidocyte, consisting of a cell wall, capsule wall, folded tubule, and enclosed operculum. (B–C) After triggering the cnidocil, the operculum opens, and the shaft with stylus is discharged into the prey’s cuticle. (D) The cnidocyte with a totally everted tubule. Drawings based on schematic drawings and high-speed video images in [29]. Scale bar 15 μm.

The eversion catapult is a mechanism referring to the discharge process of the cnidocyte: a specialized explosive stinging cell used for prey capture, anti-predator defense, and locomotion (Fig 22A) [29,97–103]. The cnidocyte is the distinguishing feature of Cnidaria, an ancient phylum of aquatic animals (>500 million years old) that includes jellyfish, sea anemones, and corals [104]. Three main types of cnidocytes are identified: (1) spirocysts, which are used to immobilize prey as they surround and adhere to prey; (2) ptychocysts, which are used by sea anemones to construct the tube in which they live; and (3) nematocysts, the most diverse group of cnidocytes, of which at least some are associated with penetration and the injection of venom [29,103,105]. Each cnidocyte contains an organelle called cnida. The cnida comprises a bulb-shaped capsule with a multi-layered [106,107] collagenous wall and a lid (operculum) that closes the capsule [100,108]. Attached to the capsule is an inverted shaft with a highly folded 3–100-μm long hollow tubule (mass of 2.3 ng; accelerated mass approximately 1 ng [2]) containing a thorn-like stylus of varying morphology between species [106–110]. Within the cnida, regulation of osmolytes leads to water absorption, which increases the osmotic pressure up to 15 MPa [102,108]. This water absorption subsequently expands the volume of the cell and stretches the elastic collagenous capsule wall [2,29,101]. When the sensory receptor (cnidocil) of the cell is triggered by either physical or chemical stimuli (e.g. vibrations or light changes) [97,98,100,101], the operculum opens, and the shaft with the tubule everts [2,97,101,104,106–109] and is discharged from the cell with a peak launch acceleration of 5,413,000*g* and a peak launch velocity of 37.1 m/s (mean: 18.6 m/s) [2,29] (Fig 22B and 22C). Next, the shaft and stylus punch a hole into the prey’s cuticle or skin of 100–600 μm in depth in jellyfish [101], with an acceleration force of 13.2–53.1 μN, kinetic energy of 0.17–0.7 μJ, and a theoretical penetration pressure of nearly 8 GPa, which is in the range of bullets [2,29] (Fig 22B–22D). The high penetration pressure is partly due to the small surface of the stylus. Based on the acceleration and velocity data, we estimated a peak mass-specific power output of shooting mechanism of 1.97·10^**9**^ W/kg, which is about 1.75·10^**6**^ times the maximum mass-specific power output of muscles [23,24], indicating the use of an elastic enhancement mechanism for the shooting action. Finally, the tubule completely everts under the built pressure and releases toxic substances into the prey or foe.

### Muscle Contraction

#### Elastic Energy Storage in Collagen Fibers—Collagen Relaxation Release Mechanism: Muscle-Powered Squeeze Catapult

The use of a prehensile tongue for capturing prey is a typical feature of the three main iguanian lizard families Iguanidae, Agamidae, and Chamaeleonidae (phylum: Chordata, class: Reptilia, order Squamata) [111]. Although all iguanian lizards use their tongue to capture prey, there are differences in the mechanism and the maximum protrusion and projection distances of the tongue. Three mechanisms of tongue protrusion are observed: (1) the tongue may undergo hydrostatic elongation, (2) the tongue may be drawn anteriorly out of the mouth by the M. genioglossus (i.e. a strong tongue muscle running from the tongue to the anterior part of the lower jaw, the chin), and (3) contraction of the M. verticalis (i.e. an intrinsic tongue muscle found at the borders of the anterior part of the tongue) surrounding a stiff bone called the entoglossal process may cause the tongue to slide forward [112]. As only the third mechanism of tongue protrusion can contain a ballistic phase, we will only discuss this mechanism. It must be noted, however, that the different mechanisms of tongue projection in iguanian lizards are not mutually exclusive and may be combined to create a variety of tongue movements.

**Fig 23:**
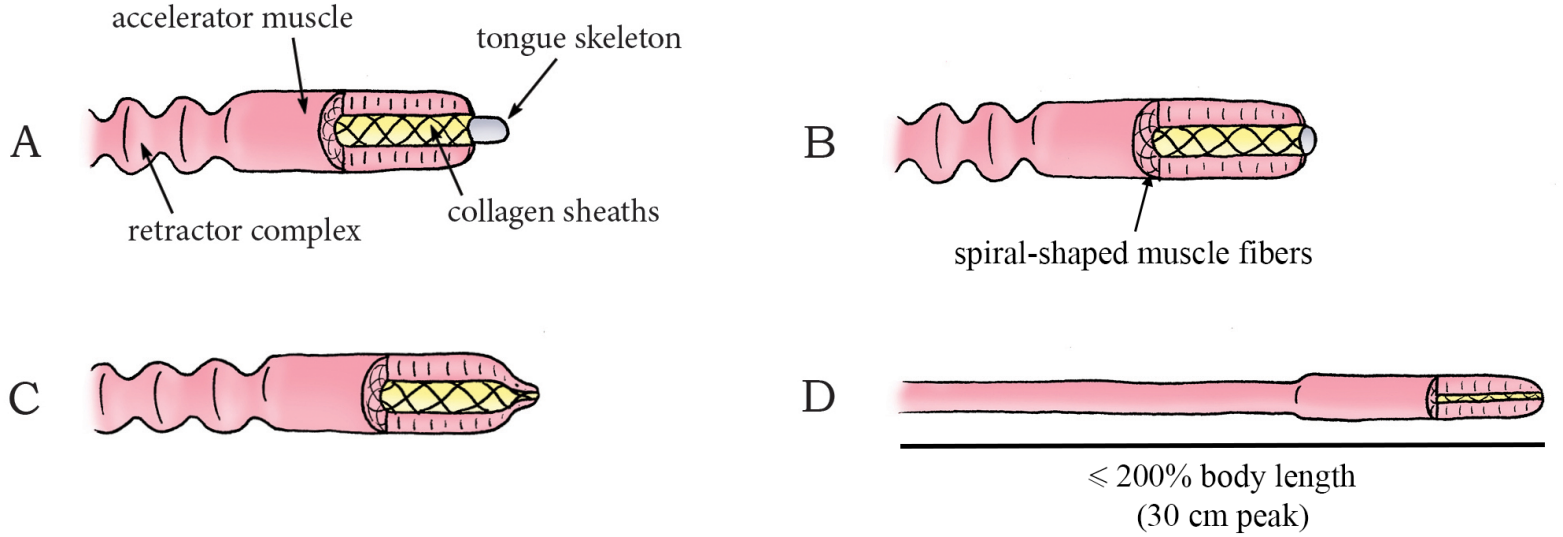
The muscle-powered squeeze catapult mechanism in the family Chameleonidae or chameleons (species *Chameleo pardalis*). (A) The chameleon tongue consisting of the tongue skeleton (entoglossal process), accelerator and retractor muscles, and nested collagen sheaths. (B) Activation of spiral-shaped muscle fibers in the accelerator muscle leads to radial contraction and elongation of the muscle and stretches the helically wound collagen fibers in the sheaths. (C–D) The accelerator muscle and sheaths slide off the tip of the bone, releasing the stored elastic energy, and forcing the tongue forward. Drawings based on schematic drawings in [30]. Scale bar indicates a length of up to 200% body length (peak distance of 30 cm in *Chameleo calyptratus* [30]).

Chamaeleonidae (phylum: Chordata, class: Reptilia, order: Squamata, suborder: Iguania), a family of old-world lizards known as chameleons, are a distinctive and highly specialized clade of over 200 described species that are found in warm habitats such as rain forest, savannas, and deserts, with various species occurring in Africa, Madagascar, Southern Europe, and Southern Asia. They are known (amongst others) for their ballistic tongue, which they use for ambushing and catching prey. For this purpose, they use a muscle-powered squeeze catapult that consists of a slender cylindrical tongue bone, the entoglossal process, surrounded by thin nested sheaths with helically wound collagen fibers (in clock-wise and anti-clockwise directions) [30] and a peripheral tubular accelerator muscle with spiral-shaped muscle fibers that are oriented perpendicular to the longitudinal direction [113] (Fig 23A). The accelerator muscle is activated approximately 200–300 ms prior to discharge in *Chameleo jacksonii* [114]. Contraction of the muscle fibers in the accelerator muscle leads to an inwardly directed normal force on the sheaths and a longitudinal pressure gradient in the muscle due to volume conservation [30]. Radial contraction of the accelerator muscle and its concomitant elongation (due to volume conservation) stretches and tensions the collagen fibers in the sheaths between the muscle and bone, primarily by the elongation of the sheaths [26,30] (Fig 23B). The stretched collagen fibers store elastic energy and exert inwardly directed normal stresses on the underlying bone. At a certain elongation of the accelerator muscle and sheaths, the most anterior collagen fibers in the collagen sheaths slide over the tip of the tongue bone, which starts the tongue projection. The force exerted by the stretched sheaths on the tongue tip results in a longitudinal (axial) reaction force of the bone on the tongue pad, which accelerates the pad forward [113]. This leads to a sliding motion of the sheaths and a sequential push off from the tongue tip of the entire sheaths (cf. ‘sliding spring theory’ [113]). The motion of the sheaths over the tongue tip reduces their diameter locally, allowing the helically arranged collagen fibers to shorten and thus release the stored elastic energy [30,113,115]. This elastic energy is directly converted into kinetic energy of the tongue pad. Frictional losses are thought to be very low due to the smooth inner and outer surfaces of the innermost sheath and the tongue bone, respectively, and the presence of a lubricant. Recently, the elastic mechanism has been modeled by Moulton *et al*. [116]. The tongue is projected forward up to 200% of the snout-vent (or body) length until it contacts the prey, such as a locust [30,115,117] (Fig 23C and 23D). The actual projection distance depends on initial prey distance and species. A highly viscous mucus on the tip of the tongue [118] and shape-adaptable concavity of the anterior pad [117] prevent the prey from escaping at this point. Finally, the tongue is retracted by the retractor muscles (Fig 23A) [119]. The launch acceleration, velocity, and power output depends on species, with the higher values measured (using high-speed video cameras or Doppler radar [120]) in the smaller species [3,121]. The maximum reported acceleration is 264*g*, observed in *Rhampholeon spinosus* [3], and the maximum reported peak launch velocity is 6 m/s, observed in *Chameleo melleri* [30]. Most mass-specific power output values for tongue projection in chameleons exceed 3,000 W/kg [3,30], with a maximum of 14,040 W/kg in *R*. *spinosus* [3], indicating that an elastic-recoil mechanism is used to enhance power output during the projection phase. This elastic mechanism allows chameleons to perform consistently in a large temperature range [26,27].

**Fig 24:**
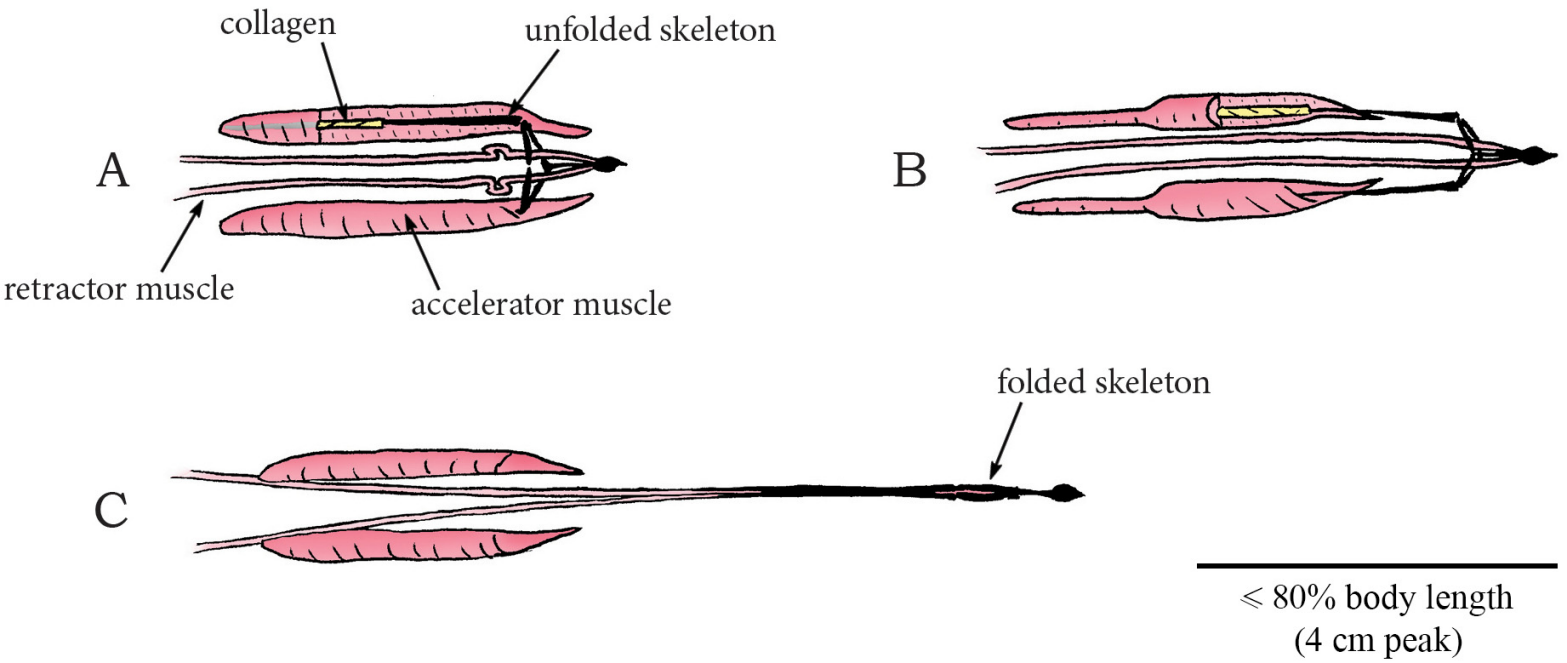
The muscle-powered squeeze catapult mechanism in the family Plethodontidae or lungless salamanders (species *Hydromantes supramontis*). (A) The tongue with the unfolded tongue skeleton, two accelerator (or protractor) muscles, collagen fibers in the sheaths between the accelerator muscles, and retractor muscle. (B) Contraction of the accelerator muscle loads the sheaths with elastic energy and forces the two posterior ends of the skeleton forward. (C) Folding of the tongue skeleton to the midline during discharge. Drawings based on schematic drawings in [127]. Scale bar indicates a length of up to 80% of the body length (peak launch distance 4 cm in *Hydromantes genei* [31]).

Plethodontidae (phylum: Chordata, class: Amphibia, order: Urodela), known as lungless salamanders, form the largest family of salamanders, with over 380 species described. Most plethodontid species are located in damp regions on the Western Hemisphere, with species found in British Columbia, Brazil, Southern Europe, and South Korea. Since Plethodontidae lack lungs, they rely on respiration through their skin and tissues lining their mouths. Ballistic tongues have evolved in at least three different clades of the lungless salamanders [31]. Their ballistic tongues are used to catch elusive prey such as flies [122]. The description given below is typical for the situation in the genera *Hydromantes* and *Bolitoglossa*. They use a muscle-powered squeeze catapult mechanism which might be considered as the inverse version of the mechanism employed by chameleons [123]. In the lungless salamanders, the tongue skeleton is made of seven flexible, interlinked cartilages, forming a fork with two posteriorly pointing teeth [117,124,125]. Each tooth (the ‘epibranchial cartilages’) of the fork is surrounded by a connective tissue sheath and the protractor muscle (the M. subarcualis rectus, the function of which corresponds to that of the accelerator muscle in the chameleon), which in turn is connected to the body of the salamander [123,126] (Fig 24A). The protractor muscles are activated approximately 123 ms prior to launch in *Hydromantis imperialis* [123]. Their contraction presumably stretches the collagen fibers in the sheaths between each of the protractor muscles and the tongue skeleton, loading the sheaths with elastic energy. The protractor muscles and sheaths drive the posterior ends of the skeleton forward (Fig 24B) [123,126]. The tongue skeleton is guided through a tractrix-shaped track in the mouth formed by the lateral wall of the cavity of the retractor muscles [125]. At a critical pressure (delivered by the protractor muscles), the tongue skeleton folds towards the midline as it slides forward through the tractrix-shaped track, forming a thin elongated lingual skeleton that is shot towards prey [122,124,126] with a peak launch distance of 80% of the body length in *Hydromantes* [117,122–124,127] (Fig 24C). A peak launch acceleration of 458*g* and a peak launch velocity of 7.0 m/s have been observed in *Bolitoglossa dofleini* [31]. The majority of the mass-specific peak-power output values for tongue projection in lungless salamanders exceed 2,000 W/kg, with a measured maximum of 18,129 W/kg in *B*. *dofleini* [31,128], indicating that an elastic-recoil mechanism is used to enhance power output [128]. Furthermore, tongue projection performance is maintained over a large temperature range (2–24°C), corroborating the presence of a catapult mechanism [128]. After the sticky tongue pad has contacted the prey, with a force between 0.026–0.44 gram depending on the distance to the target [126], it is rapidly retracted and the mouth is opened further to make room for the tongue and prey [122,124,128]. Finally, the mouth closes, enwrapping the prey. Thus, in the lungless salamanders, the tongue skeleton is accelerated off the muscles and projected towards the prey, whereas in chameleons the soft parts of the tongue are accelerated off the entoglossal process. The catapult mechanism that evolved in both these disparate clades is nevertheless very similar. The different tongue architectures are presumably the result of different initial conditions of the evolutionary process that yielded these highly effective prey capture devices.

#### Elastic Energy Storage in Saddle-Shaped Structure—Latch Release Mechanism: Linkage and Latch Catapult

**Fig 25:**
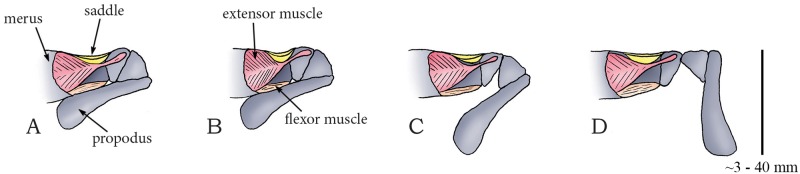
Linkage and latch catapult mechanism in the phylum Stomatopoda (species *Odontodactylus scyllarus*). (A) The appendage of the mantis shrimp consisting of a saddle-shaped elastic structure located between the merus and the propodus. (B) Compression of the saddle and latching of the propodus by the simultaneous contraction of the extensor and flexor muscles. (C–D) Release of the latch by relaxation of the flexor muscle, allowing outward movement of the propodus. Drawings based on schematic drawings in [133]. Scale bar indicates the length of the propodus, which is in between 3 and 40 mm.

Stomatopoda (phylum: Arthropoda, class: Malacostraca), commonly known as mantis shrimps, is an order of marine crustaceans consisting of over 450 species. Mantis shrimps come in a variety of sizes, from a few centimeters up to 40 centimeters, and colors, from brown to several vivid colors in the peacock mantis shrimp (*Odontodactylus scyllarus*). They can be found in shallow, tropical and subtropical marine habitats. These solitary crustaceans spend most of their time hiding in rock formations or burrows, from where they either ambush prey by sitting and waiting for prey to chance upon them, or hunt, chase, and kill prey using a fast appendage strike, which produces forces of up to 1,500 N depending on appendage size [129,130]. The appendage consists of a saddle-shaped elastic structure located between the base (or merus) and the flexing part (or propodus) of the appendage (Fig 25A). This saddle-shaped spring is compressed and latched by the simultaneous contraction of the extensor and flexor muscles (Fig 25B) [131]. When the flexor muscle relaxes, the latch is released, allowing the appendage to rotate outwards (Fig 25C and 25D) [129–133] with a maximum reported peak launch acceleration of 10,601g and a peak launch velocity of 23 m/s observed in *O*. *scyllarus* [25,131]. Based on this data, a mean power output of 470,000 W/kg is conservatively calculated in *O*. *scyllarus* [25]. Furthermore, *O*. *scyllarus* and some other species of mantis shrimps are so fast that they are able to create cavitation bubbles that can aid to stun prey by generating shockwaves, bright light flashes, and rapidly heat the water, when they implode [25,130].

#### Elastic Energy Storage—Unknown Release Mechanism: Inertial Elongation Catapult

**Fig 26:**
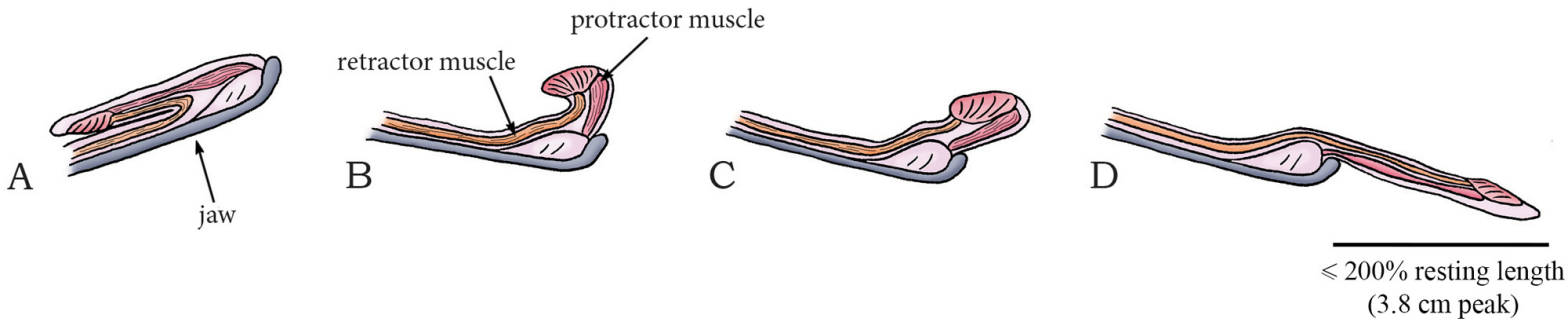
Inertial elongation catapult mechanism in the order Anura (species *Bufo marinus*). (A) The jaw and tongue of frogs and toads using the inertial elongation catapult in rest position. (B) Contraction of the protractor muscle moves the tongue up- and forward. (C–D) Rapid jaw depression accelerates and elongates the tongue using the tongue’s own inertia. Drawings based on schematic drawings in [16]. Scale bar indicates a length of up to 200% of the resting length (peak launch distance 3.8 cm in *Rana pipiens* [139]).

Early studies of the feeding system in anurans (frogs of the phylum Chordata, class: Amphibia) identified three basic mechanisms of tongue protraction: hydrostatic elongation, mechanical pulling, and inertial elongation [134,135]. As only the third mechanism contains a ballistic phase, only this mechanism will be discussed. The inertial elongation catapult is probably the most prevalent mechanism of tongue protraction among anurans (frogs of the phylum: Chordata, class: Amphibia) [134] and is characterized by tongue lengthening under inertial and muscular loading using a rapid jaw depression [16,134–136]. It has evolved at least seven times independently in the families Bufonidae (of which *Bufo* is the most widespread genus) [16], Hylidae (including the genera *Pachymedusa* and *Phyllomedusa*) [16,137], Microhylidae (including the genus *Dyscophus*) [138], Dendrobatidae, Megophryidae, Leptodactylidae, and Ranidae (including the genus *Rana*) [135,136,139,140]. The tongues of most frogs and toads in these families are attached anteriorly at the front of the jaw and have a resting length of approximately the size of the jaw [117] (Fig 26A). During tongue protrusion, the tongue initially shortens by contraction of the protractor muscle, moving the tongue up- and forward (Fig 26B). Subsequently, a rapid jaw depression elongates and moves the tongue downwards under inertia to catch elusive prey [16,136,138,139,141] (Fig 26C and 26D). The maximum reported tongue acceleration is 31.6*g* and the maximum reported peak tongue velocity is 2.9 m/s, both observed in *Bufo marinus* [136]. After the prey is caught, the retractor muscle retracts the tongue, while the mouth is opened further to accommodate the prey (Fig 26B) [139].

There are several indications that elastic recoil is used in the rapid jaw depression. Firstly, the jaw depressor muscles are active prior to the onset of jaw depression, pointing towards an energy-storing phase [142]. Secondly, the ballistic tongue protrusion is not influenced by temperature changes (tested between 10 and 35°C), yet pronounced differences are visible for tongue retraction and mouth closing [139,143]. Thirdly, the power of ballistic mouth opening reaches a peak of 9,600 W/kg [142] and 1,783 W/kg (885 W/kg for tongue protrusion) [139] in *B*. *alvarius* and *Rana pipiens*, respectively, exceeding what amphibian muscles can directly produce [22] by more than 10 times in the case of *B*. *alvarius* and by almost 5 times in *R*. *pipiens*.

#### Unknown—Eversion Release Mechanism: Muscle-Powered Eversion Catapult

**Fig 27:**
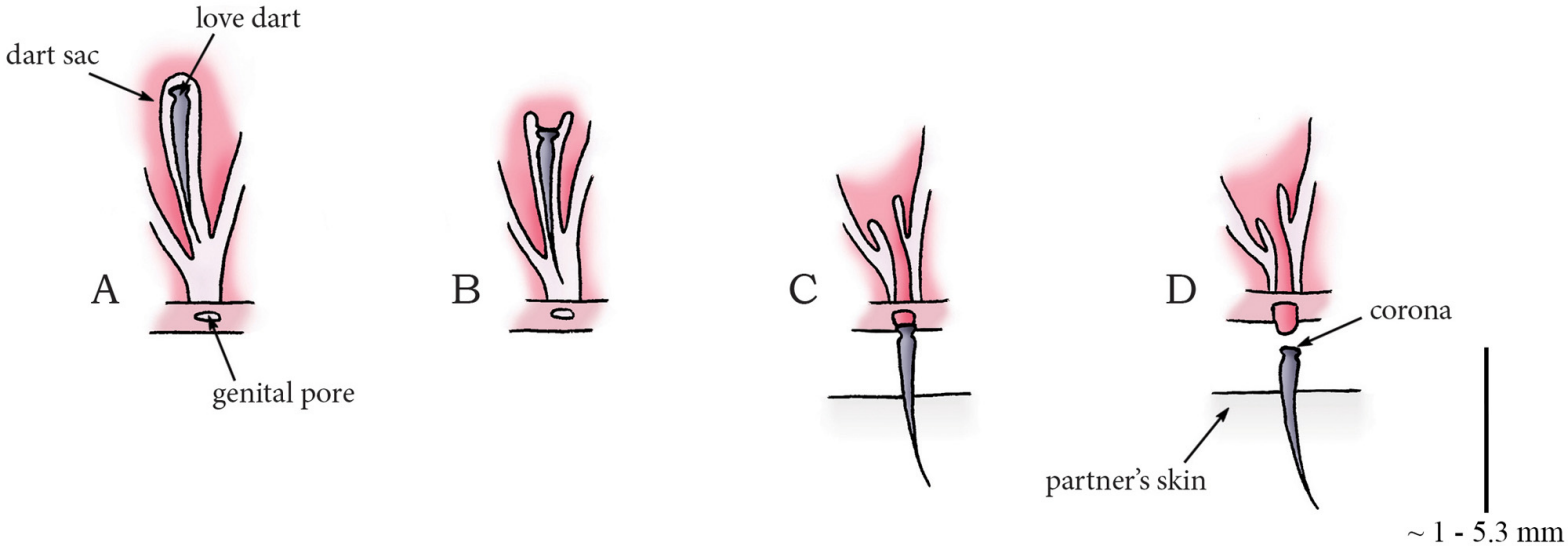
Muscle-powered eversion catapult mechanism in the clade Stylommatophora (species *Cornu aspersum*). (A) The dart organ morphology, consisting of a fully developed love dart and a muscular dart sac. (B) Eversion of the dart sac, forcing the love dart to be externalized through the genital pore. (C–D) Piercing and release (at the corona) of the love dart into the mating partner. Drawings based on schematic drawings in [145]. Scale bare indicates the length of the love dart, which is in between 1–5.3 mm in length (longest dart found in *Chilostoma cingulatum* [146]).

In many terrestrial snails and slugs, a relatively strange mating process can be observed in which both mating partners try to shoot a calcareous (love) dart out of the genital pore into the skin of “unlucky” mating partner [144]. Dart shooting is present in at least 9 families; the Ariophantidae, Bradybaenidae, Dyakiidae, Helicidae (including the genus *Cornu*), Helminthoglyptidae, Hygromiidae, Parmacellidae, Urocyclidae, and Vitrinidae, within the clade Stylommatophora (phylum: Mollusca, class: Gastropoda), which comprises 60 families of snails and slugs. The darts consist of a corona of pleated blades that connect the dart to the body of the snail, a concave connection of the corona to the main shaft of the dart, called the flare, and a shaft with multiple vanes [145] covered in mucus that reconfigures the female reproductive system and allows for more sperm to fertilize the eggs; increasing the dart shooter’s paternity [144,146,147]. Darts exhibit large diversity, with some species having simple cone-shape darts and others having curved or contorted darts [146]. Surrounding the darts is the so-called dart sac. Dillaman [145] and Koene *et al*. [147] described the dart sac as a muscular organ with layers of connective tissue surrounding mainly the posterior end of the dart (near the corona). Unfortunately, the description of the shooting action is limited to a claim that the dart is externalized by the eversion of the dart sac (Fig 27A–27D) [144,146]. Based on dart sac morphology, one may assume that the dart sac muscles with connective tissue drive the eversion upon activation. However, additional research is warranted to determine the working principle of this dart shooting mechanism, as well as the associated launch parameters.

## Discussion

### Energy Generation, Storage, and Transformation

This systematic review provides an overview of the working principles of shooting mechanisms found in nature with a focus on the energy management prior to and during the shooting action. In fungi, energy for shooting is generated via osmosis in the form of either water condensation on the system, water absorption into the system, or water evaporation from the system. In plants, energy for shooting is also generated via osmosis, in the form water absorption into the system or water evaporation from the system; the water condensation mechanism is not observed. In animals, the energy for shooting is mostly produced by muscles, with the exception of the phylum Cnidaria, in which energy is generated via osmosis (water absorption specifically).

In all identified shooting mechanisms in plants and fungi, the generated energy is gradually stored as elastic energy using specialized wall structures, often comprising several cell layers, with two exceptions: the momentum catapult and the air pressure catapult. In the former, the energy for shooting is stored in the sterigma by a shift in the center of mass, whereas in the latter, energy is primarily stored in the compressed air trapped inside the containers (apart from energy stored in stored in the cell walls). In animals, the generated energy is gradually stored in elastic structures such as collagen or resilin. The use of elastic structures is advantageous when high launch accelerations and velocities are desired, as elastic structures can recoil much faster than muscles can shorten [20,21], which leads to a higher power output during the launch of the projectile. Furthermore, the power release of these elastic structures is less temperature-sensitive than that of the muscles (because the work production by the muscles is much less temperature dependent than the power output), allowing the animals to perform over a wide temperature range [26–28]. However, albeit “slow”, the non-elastically enhanced hydrostatic and directly muscle-powered tongue protrusion mechanisms found in anurans [16] and iguanian lizards [112] allow for a better control of the direction of motion of the tongue during protrusion, which is very hard (if not impossible) in elastically enhanced shooting mechanisms.

The trigger for the release of the energy needed to discharge the projectile differs greatly between shooting mechanisms. Fracture of the cell walls and the development of gas bubble(s) within the structure surrounding the projectile are common triggers in plants and fungi. Another trigger found in fungi is the eversion of the structure (e.g. membrane) surrounding the projectile. Eversion as a release mechanism is also encountered in animals, in the phylum Cnidaria and the clade Stylommatophora (snails and slugs), specifically. In order to trigger the eversion of the structure or projectile, a generally small “energy bump” needs to be overcome which prevents an uncontrolled release. This energy can possibly be delivered by a slight outwards movement of the cup or cnida, which straightens the membrane or shaft in *Sphaerobolus* and Cnidaria, respectively. In the family Chameleonidae (chameleons), elastic relaxation of the collagen sheaths as they slide off the tongue bone is used as trigger in tongue shooting, whereas in Plethodontidae (lungless salamanders) the elastic energy is released when the forked lingual skeleton folds medially by the tractrix-shaped muscle track; allowing the collagen sheaths to propel the tongue bone out of the mouth and relax. Unfortunately, the release mechanism in the inertial elongation catapult in anurans (frogs and toads) has not been fully unraveled yet. It may be questioned whether reloadability of the shooting mechanism compromises launch velocity and acceleration.

In non-reloadable shooting mechanisms such as those in fungi and plants (which release energy by fracture, cavitation, or eversion), mechanical stresses can be increased upon failure of the cells. In reloadable systems such as most mechanisms in animals (except for the osmotic-powered and muscle-powered eversion catapult), mechanical stresses should be kept below the threshold of permanent tissue damage, so that complete reloading of the shooting mechanism is possible. In other words, the performance of shooting mechanisms in animals that are used for prey capture might be constrained by the reusability requirement.

Directly after the release trigger, the projectiles are discharged. A variety of catapult mechanisms, such as the coiling catapults, squeeze catapults, and muscle-powered eversion catapults, are identified that transform the (stored elastic) energy into kinetic energy of the projectile. For the small untethered projectiles, such as spores, seeds, and pollen, present in fungi and plants, the adhesion and surface tension that binds them together and to their support must be overcome during this phase [33], which in turn may result in the need for high launch velocities and accelerations to reach sufficient launch distances. Projectiles have been identified ranging from 10^−9^ mg (spore mass) in Ascomycota and Zygomycota to approximately 10,300 mg (tongue mass) in *B*. *alvarius*. Tables 1, 2 and 3 show that the highest accelerations are found in the launch of microscale projectiles, with peak accelerations of up to 5,413,000*g* in cnidarians (projectile mass of approximately 1·10^−6^–2.3·10^−6^ mg) [2] and 870,000*g* in *G*. *zeae* (projectile mass of approximately 0.2·10^−6^ mg) [21]. For larger projectiles, such as the tongue in lungless salamanders (tongue mass of approximately 1,000 mg), peak accelerations reach “only” 458*g* in *B*. *dofleini* [31]. Why is this the case? According to Newton’s second law (force = mass times acceleration), a smaller projectile mass can be given an equal acceleration with a lower force. However, mass increases with length cubed, whereas force tends to increase with cross-sectional area and thus length squared. On a small scale, drag forces become more dominant; consequently, micrometer-scale projectiles are decelerated in their free flight almost instantaneously after discharge. In other words, for the same initial launch velocity and acceleration, larger projectiles travel farther than smaller ones if the effect of external flow (e.g. wind) is not taken into account [34].

Depending on the main function of the shooting mechanism, e.g. food capture or seed dispersal, different characteristics of the shooting mechanism are of importance, such as the launch velocity, launch acceleration, dispersal distance, spread, and accuracy. In plants and fungi, the dispersal distance and spread are the most important parameters. The effectiveness of the shooting mechanisms in terms of improving survival of the spore or seed can be mainly deducted from the dispersal patterns [69]. Seed or spore dispersal over even a short distance from the plant (beyond the canopy) or fungus can already increase the probability of seed or spore survival. However, high dispersal distances and spread tend to increase the survival rate and reproductive success [77,86,94]. Furthermore, by projecting spores into an airstream (wind), fungi or mosses can increase the probability of encountering susceptible hosts or environments [10]. To negate the negative effects of viscous drag on the launch distance, plants and fungi often synchronize the discharge of thousands of (often small) pollen, seeds, and spores, such as in ascomycetes [46], *Sphagnum* [1], and *S*. *martensii* [96], or optimize the shape of the projectile [45]. In animals, on the other hand, high launch acceleration, launch velocity, and accuracy are needed, as the shooting mechanisms are critical for territory and self-defense, prey capture, substrate attachment, and locomotion. For example, in chameleons and lungless salamanders, the tongue is critical for catching elusive prey, and in cnidarians the cnidocysts are important for self-defense and locomotion (amongst others) [97]. A trade-off seems to be present between the launch velocity (and acceleration) and accuracy, with faster shooting mechanisms being less accurate. For example, in anurans the inertial elongation catapult mechanism has an accuracy of approximately 33%, whereas the non-elastically enhanced and much slower hydrostatic mechanism has an accuracy of over 99% [16]. This difference in accuracy can be mainly led back to the lack of feedback control (i.e. the inability to adjust the trajectory during the shooting action) of the inertial elongation catapult and muscle-powered squeeze catapult.

### Power output, Work, and Scale Effects

The scale of the shooting mechanisms, in particular the mass of the projectile, has been shown to affect the launch acceleration and velocity of the projectile. If we look into the power output per unit mass, it becomes clear that the highest values are found in the shooting mechanisms with projectiles that have a size at microscale. For example, the shooting mechanisms identified in *C*. *canadensis* and *C*. *parviflora*, with projectile masses of 0.024 mg and 0.15 mg, respectively, have an power output that is more than 46 times higher than that of *C*. *melleri* and *B*.*dofleini*, with a tongue mass of 4,000 mg and 1,000 mg, respectively (see Tables 2 and 3). Furthermore, Anderson [3] found that smaller chameleons, such as *R*. *spinosus* (12,100–14,040 W/kg), outperform larger species, such as *Furcifer oustaleti* (1,410–2,980 W/kg) in terms of power output per unit mass. The highest identified power output found is in cnidarians (1.97·10^9^ W/kg), which shoot their stinging cells with a mass of in between 1–2.3 ng.

We also investigated the work per unit mass (J/kg; calculated by integrating the power output per unit mass over the launch duration) delivered by the shooting mechanisms (see Tables 1–3). It appears that the work per unit mass is largely independent of the scale of the mechanism. For example, similar work per unit mass values have been calculated in the microscale shooting mechanism of the fungus *P*. *kleinii* (sporangiophore length 2 mm; 201.5 J/kg) and the tongue shooting mechanism of the much larger chameleon *Trioceros cristatus* (with a body length of 25–28 cm; 191.8 J/kg). If we look into the difference between osmotic-powered (0.3–4,137 J/kg) and muscle-powered mechanisms (0.4–1,269 J/kg), higher values of work per unit mass are observed in the mechanisms using osmosis as energy source. The scale-dependent power output and scale independent work per unit mass per shooting mechanism substantiate that the smaller shooting mechanisms are able to release the energy for shooting faster than the larger mechanisms. However, additional research is necessary to determine the work per unit mass more precisely per shooting mechanism, as our calculations give an approximate value based on the launch duration and power output value.

### Limitations of This Study

A systematic approach was undertaken to maximize the chance of identifying relevant shooting mechanisms in nature. However, chances still remain that relevant shooting mechanisms have been overlooked by, for example, missing relevant keywords, by only including literature that was published in the English language, and by not searching in grey literature. Moreover, the lack of a full description of the biomechanical structure and activity of the complete elastic enhancement system in some of the shooting mechanisms have led to uncertain placement of these mechanisms in the classification schemes. Finally, for many of the described shooting mechanisms, one or more of the launch parameters, such as those of the cavitation coiling catapult of fungi imperfecti, and information about the exact working principle, such as that of *Viola*, were not found.

### Implications for Future Research & Applications

Future studies could be focused on supplementing the current knowledge of the working principles, including a full description of the cell structure and morphology of the shooting mechanism, and launch parameters of many of the described shooting mechanisms that are currently missing, such as the shooting mechanism in love dart shooting snails. Furthermore, some of the launch parameters can be determined with greater accuracy by using modern technology such as high-speed cameras. Additionally, shooting mechanisms that involve a liquid projectile, such as used in archerfish [11], and elastically enhanced movements, such as the Venus Flytrap [8], could be investigated in more detail based on energy management during the rapid movements. Insight into these mechanisms may point towards still unidentified working principles of shooting in nature.

The described shooting mechanisms can be used in a biomimetic approach to develop faster and smaller artificial shooting mechanisms. Opportunities can be found in applications requiring accurate cutting, separation, or connection of (e.g. soft, slippery, or elastic) materials, high forces or accelerations, as well as slender structures that suffer from buckling (a typical failure mode in percutaneous tools, such as guidewires, catheters, and needles, and slender industrial tools, such as nails and needles) [148,149].

## Conclusions

In this paper a structured overview of biological projectile shooting mechanisms is provided. The reviewed shooting mechanisms are described based on how energy is managed prior to and during the shooting action, that is, how the energy is generated, stored, and transformed to kinetic energy of the projectile. Two main energy sources are identified: osmosis (in plants, fungi, and animals) and muscle contraction (in animals). The generated energy is gradually stored in an elastic structure, and transformed into kinetic energy of the projectile using a variety of release, trigger, and catapult mechanisms. The launch parameters were found to be mainly dependent on the size of the projectile, with smaller projectiles being launched at higher accelerations and velocities. The highest identified launch acceleration is 5.41·10^6^*g*, observed in cnidarians, and the highest velocity is 237 m/s, observed in the mulberry *M*. *alba*. These high accelerations are a necessity to partly negate the effects of viscous drag on small reproductive units in fungi and plants. Furthermore, in smaller shooting mechanisms discharging projectiles on microscale, higher mass-specific power outputs (up to 1.97·10^9^ W/kg in cnidarians) are observed, meaning that the smaller mechanisms are able to release the stored elastic energy faster than larger ones. This becomes especially apparent when comparing the mass-specific power output (ranging from 0.28–4,137 J/kg) with the mass-specific work, which is mostly scale-independent, with similar values in the microscale shooting mechanism of *P*. *kleinii* (201.5 J/kg) and the tongue shooting mechanism of the much larger chameleon *Trioceros cristatus* (191.8 J/kg). However, osmotic-powered shooting mechanisms seem to be able to store more energy per kilogram (≤ 4,137 J/kg) than muscle-powered mechanisms (≤ 1,269 J/kg). The given insights into the working principles improves the understanding of how nature is able to exhibit extreme launch parameters, can aid in unraveling the working principles of other, less researched, shooting mechanisms, and can potentially be used as inspiration for the design of faster artificial shooting mechanisms.

## Supporting Information

S1 Appendix Full search strategy. (DOCX)

S1 Checklist PRISMA checklist. (DOCX)

S1 Fig Flow chart for the identification and selection of literature about shooting mechanisms in plants and fungi. (TIF)

S2 Fig Flow chart for the identification and selection of literature about shooting mechanisms in plants and fungi. (TIF)
