# Supplementary material for: Shooting Mechanisms in Nature: A Systematic Review
Source: PLoS One. 2016 Jul 25;11(7):e0158277. doi: 10.1371/journal.pone.0158277 (PMC4959704; doi:10.1371/journal.pone.0158277)
Supplement: S1 Appendix — (DOCX) [file pone.0158277.s001.docx]

# S1 Appendix

## Plants and Fungi Search Queries

For the plants and fungi, the following search query was conducted in Scopus: TITLE-ABS-KEY ((projectile$ OR pollen OR seed$ OR *spore* OR perid* OR gleba) W/10 (shooting OR expulsion OR firing OR eject* OR catapult* OR launch* OR propel*)) OR TITLE-ABS-KEY ((explosive OR violent OR forceful) W/2 (discharge OR dispersal)) AND TITLE-ABS-KEY (organism$ OR species OR plant* OR flower* OR tree* OR vine* OR fung* OR *moss* OR grass* OR weed* OR herb*) AND NOT TITLE-ABS-KEY (military OR forens* OR germinat* OR propellant OR machine*) AND NOT TITLE-ABS-KEY ((launch*) W/3 (project OR program OR experiment* OR initiative$)).

In the Web of Science Core Collection the following search query was used: (TS= ((projectile$ OR “pollen” OR seed$ OR *spore* OR perid* OR “gleba”) NEAR/10 (“shooting” OR “expulsion” OR “firing” OR eject* OR catapult* OR launch* OR propel*)) OR TS= (("explosive" OR "violent" OR "forceful") NEAR/2 ("discharge" OR "dispersal"))) AND TS= (organism$ OR “species” OR plant* OR flower* OR tree* OR vine* OR fung* OR *moss* OR grass* OR weed* OR herb*) NOT TS= (“military” OR forens* OR germinat* OR “propellant” OR machine*) NOT TS= (launch* NEAR/3 (“project” OR “program” OR experiment* OR initiative$)).

## Animals Search Queries

For the animals category, the following search query was executed in the database of Scopus: TITLE-ABS-KEY ((projectile$ OR *dart$ OR arrow$ OR cnid* OR tongue$ OR appendage$) W/10 (shooting OR firing OR striking OR strike OR projecting OR projection OR protract* OR catapult* OR launch* OR eject* OR propel*)) OR TITLE-ABS-KEY ((cnid*) W/10 (discharg*)) AND TITLE-ABS-KEY (organism$ OR species OR fauna OR zoo* OR animal* OR nonhuman OR creature$ OR genus) AND NOT TITLE-ABS-KEY (military OR forens* OR germinat* OR propellant OR machine* OR plasma OR ioniz* OR archaeology OR euthanasia OR an$esthesia OR thalam*) AND NOT TITLE-ABS-KEY ((launch*) W/3 (project OR program OR experiment* OR initiative$)) AND NOT TITLE-ABS-KEY ((tongue$ OR tal$) W/2 (flick*)).

In the Web of Science Core Collection, the following search query was used: (TS= ((projectile$ OR *dart$ OR arrow$ OR cnid* OR tongue$ OR appendage$) NEAR/10 (“shooting” OR “firing” OR “striking” OR “strike” OR “projecting” OR “projection” OR protract* OR catapult* OR launch* OR eject* OR propel*)) OR TS= ((cnid*) NEAR/10 (discharg*))) AND TS= (organism$ OR “species” OR “fauna” OR zoo* OR animal* OR “nonhuman” OR creature$ OR “genus”) NOT TS= (“military” OR forens* OR germinat* OR “propellant” OR “machinery” OR “plasma” OR ioniz* OR archaeolog* OR “euthanasia” OR an$esthesia OR thalam*) NOT TS= (launch* NEAR/3 (“project” OR “program” OR experiment* OR initiative$)) NOT TS= ((tongue$ OR tail$) NEAR/2 flick*).
