# Supplementary figures and images for: Shooting Mechanisms in Nature: A Systematic Review
Source: PLoS One. 2016 Jul 25;11(7):e0158277. doi: 10.1371/journal.pone.0158277 (PMC4959704; doi:10.1371/journal.pone.0158277)

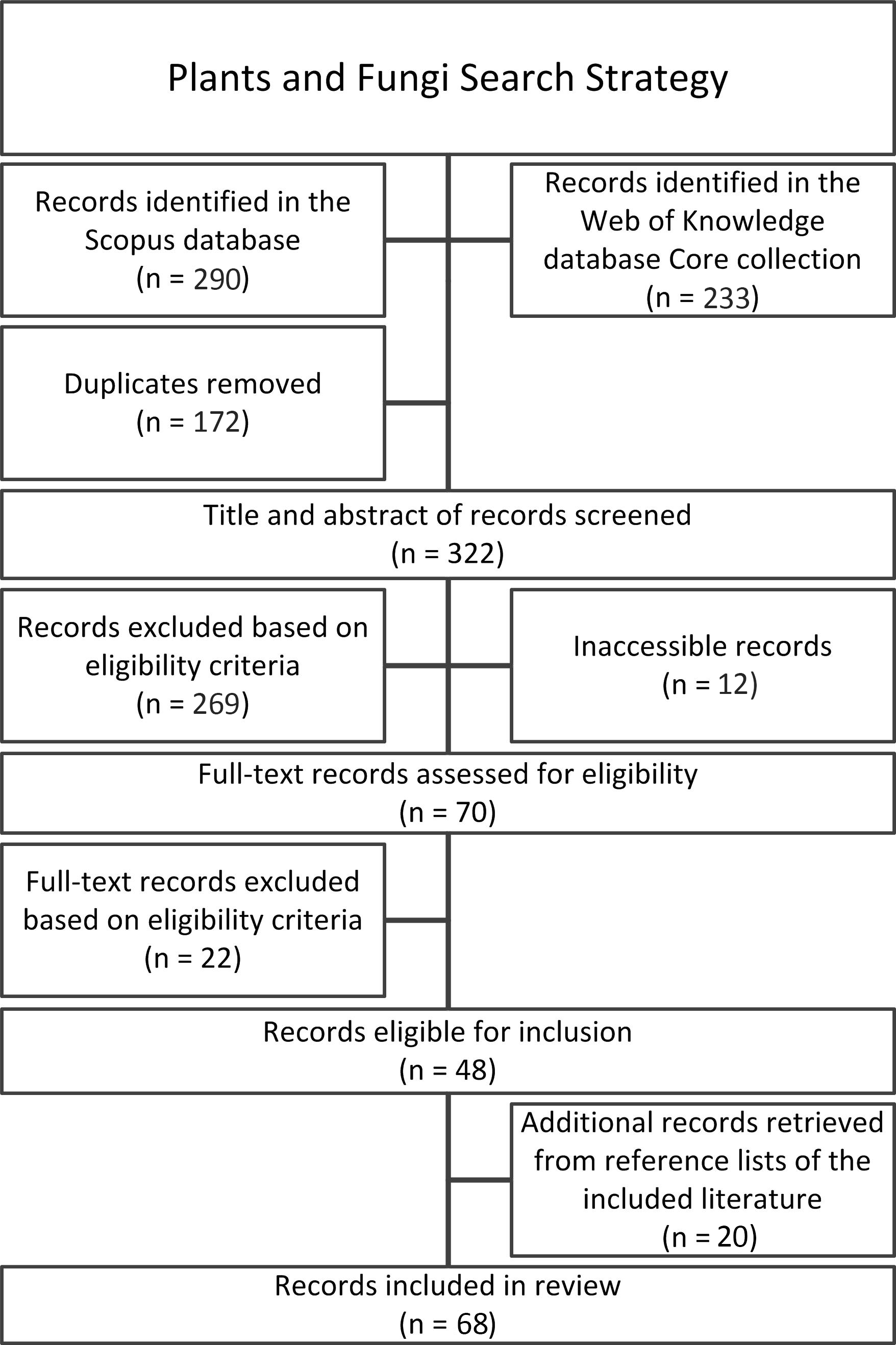

Supplement: S1 Fig — (TIF) [file pone.0158277.s003.tif]

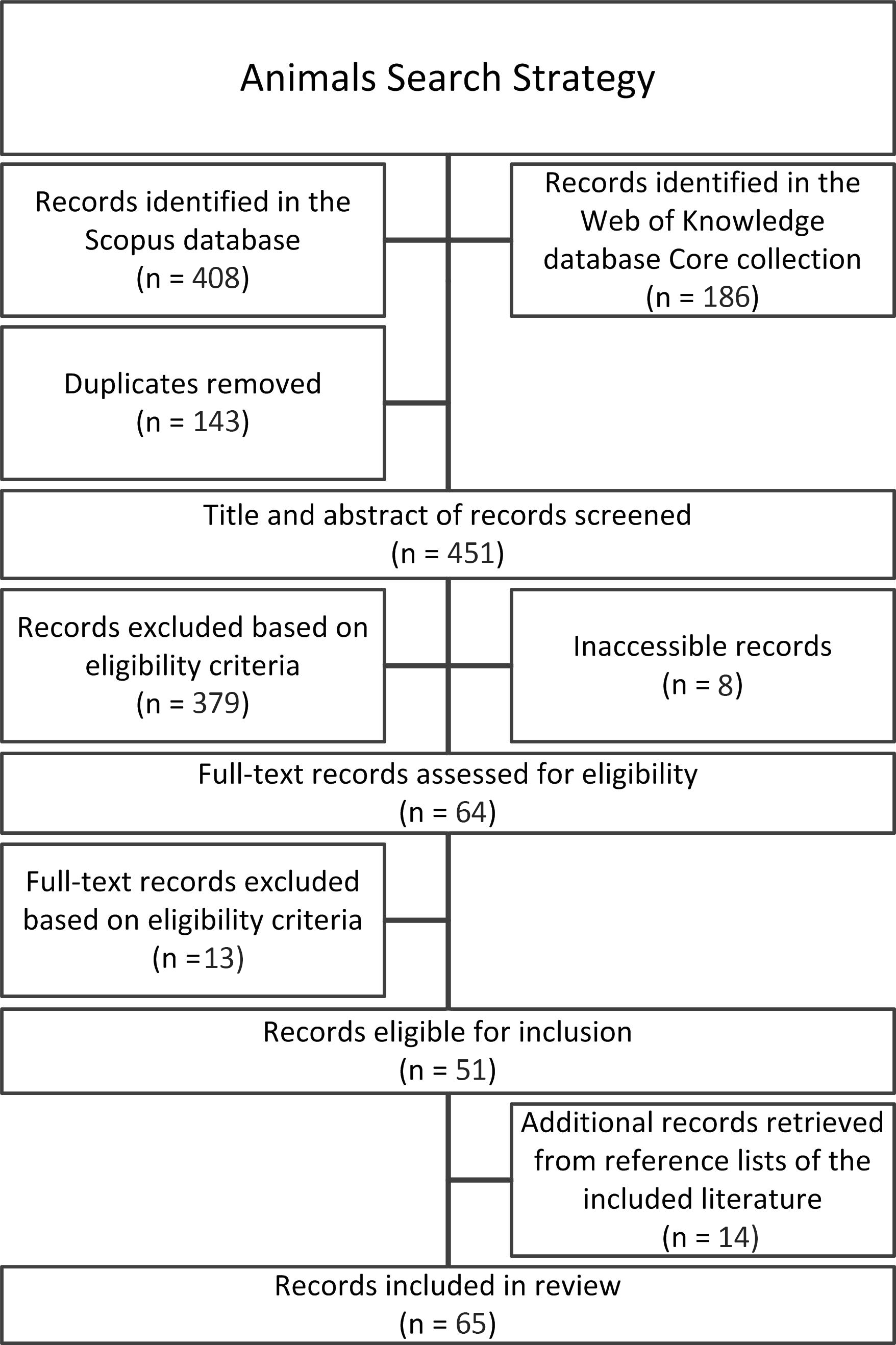

Supplement: S2 Fig — (TIF) [file pone.0158277.s004.tif]
